# Atherosclerotic cardiovascular disease risk assessment: An American Society for Preventive Cardiology clinical practice statement

**DOI:** 10.1016/j.ajpc.2022.100335

**Published:** 2022-03-15

**Authors:** Nathan D. Wong, Matthew J. Budoff, Keith Ferdinand, Ian M. Graham, Erin D. Michos, Tina Reddy, Michael D. Shapiro, Peter P. Toth

**Affiliations:** a Heart Disease Prevention Program, Division of Cardiology, University of California, Irvine, CA, United States; b Division of Cardiology, Harbor-UCLA Medical Center, Torrance, CA, United States; c Tulane University Heart and Vascular Institute, Tulane University School of Medicine, New Orleans, LA United States; d Department of Cardiology, Trinity College, Dublin, Ireland; e Ciccarone Center for the Prevention of Cardiovascular Disease, Johns Hopkins University School of Medicine, Baltimore, MD, United States; f Division of Cardiology, Wake Forest University, NC, United States; g CGH Medical Center, Sterling, IL, United States

**Keywords:** Cardiovascular disease, Risk assessment, Risk factors inflammation, Sex, Ethnicity, Subclinical atherosclerosis, Primary prevention, Secondary prevention, ASCVD, atherosclerotic cardiovascular disease, ACC, American College of Cardiology, AHA, American Heart Association, ASPC, American Society for Preventive Cardiology, BMI, body mass index, CAC, coronary artery calcium, CCTA, coronary computed tomography angiography, CHD, coronary heart disease, CKD, chronic kidney disease, CRP, C-reactive protein, CVD, cardiovascular disease, DM, diabetes mellitus, EAS, European Atherosclerosis Society, ESC, European Society of Cardiology, FH, familial hypercholesterolemia, GDM, gestational diabetes mellitus, IMT, intima media thickness, LDL, low density lipoprotein, MMP, matrix metalloproteinase, NHB, non-Hispanic Black, NHW, non-Hispanic White, PAD, peripheral arterial disease, PCOS, polycystic ovary syndrome, POI, premature ovarian insufficiency, PCE, pooled cohort equation, SDOH, social determinants of health, VTE, venous thrombotic event

## Abstract

Risk for atherosclerotic cardiovascular disease (ASCVD) shows considerable heterogeneity both in generally healthy persons and in those with known ASCVD. The foundation of preventive cardiology begins with assessing baseline ASCVD risk using global risk scores based on standard office-based measures. Persons at low risk are generally recommended for lifestyle management only and those at highest risk are recommended for both lifestyle and pharmacologic therapy. Additional “risk enhancing” factors, including both traditional risk factors and novel biomarkers and inflammatory factors can be used to further assess ASCVD risk, especially in those at borderline or intermediate risk. There are also female-specific risk enhancers, social determinants of health, and considerations for high-risk ethnic groups. Screening for subclinical atherosclerosis, especially with the use of coronary calcium screening, can further inform the treatment decision if uncertain based on the above strategies. Persons with pre-existing ASCVD also have variable risk, affected by the number of major ASCVD events, whether recurrent events have occurred recently, and the presence of other major risk factors or high-risk conditions. Current guidelines define high to very high risk ASCVD accordingly. Accurate ASCVD risk assessment is crucial for the appropriate targeting of preventive therapies to reduce ASCVD risk. Finally, the clinician-patient risk discussion focusing on lifestyle management and the risks and benefits of evidence-based pharmacologic therapies to best lower ASCVD risk is central to this process. This clinical practice statement provides the preventive cardiology specialist with guidance and tools for assessment of ASCVD risk with the goal of appropriately targeting treatment approaches for prevention of ASCVD events.

## 1 Introduction

The initial framework for atherosclerotic cardiovascular disease (ASCVD) risk assessment began with the Framingham Heart Study, the longest running study of cardiovascular disease in the world, and one with many “first” discoveries about the etiology of ASCVD. Former Framingham director Dr. William B. Kannel in 1961 coined the term “risk factors”, widely regarded as beginning the field of preventive cardiology. In his article “Factors of Risk in the Development of Coronary Heart Disease: Six Year Follow-up Experience: The Framingham Study” (1), it was described how elevated cholesterol, elevated blood pressure, and left ventricular hypertrophy predicted the subsequent development of coronary heart disease (CHD) events. Importantly, the burden of risk factors was directly related to the risk of CHD, probably the first demonstration of the concept we now call “global risk”. Framingham developed the first multivariable risk assessment equations utilizing logistic regression (2), with Dr. Kannel as early as 1976 noting that risk functions provide an “economic and efficient method of identifying persons at high cardiovascular risk who need preventive treatment” (3), laying the foundation for individualized risk assessment. The American College of Cardiology (ACC) Bethesda Conference 20 years later noted the intensity of treatment should match a person's risk (4). Since a clinician's estimate (without doing formal risk assessment) is most often inaccurate and underestimates risk (5), using global risk scores can improve the use of guideline-based therapy (6).

The objective of this American Society for Preventive Cardiology (ASPC) Clinical Practice Statement is to provide the preventive cardiology specialist with guidance and the tools for assessment of ASCVD risk. This includes global risk estimation from use of risk scoring (and where it may overestimate risk), use of traditional risk factors and novel biomarkers and inflammatory factors as risk enhancing factors, considerations for female-specific factors, race/ethnicity, and social determinants of health, the role of screening for subclinical atherosclerosis, as well as risk assessment for those with pre-existing ASCVD. Guidance is provided in relation to and to supplement existing guidelines on risk assessment. Nutritional factors, physical activity and cardiorespiratory fitness levels also have important roles in assessment of ASCVD risk and are discussed in other ASPC Clinical Practice Statements.

## 2 Cardiovascular risk scores and incorporation into cardiovascular prevention guidelines

### 2.1 Origin of global risk assessment

The first cardiovascular disease (CVD) risk scores were championed by the Framingham Heart Study for the prediction of CHD risk over 10 years (7) which assigned points in separate scales for men and women corresponding to different levels of age, total and high-density lipoprotein (HDL)-cholesterol, blood pressure, smoking and diabetes status from which the points were summed to a total that corresponded to a 10-year risk estimate for CHD. The Third Adult Treatment Panel of the National Cholesterol Education Program in 2001 (8) was the first clinical application of these risk equations for stratification of persons into low (<10%), intermediate (10-<20%), or high (>=20% or with known CHD or other CHD risk equivalents) 10-year risk of CHD for which specific treatment initiation and target levels of low-density lipoprotein (LDL)-cholesterol were recommended. Framingham also developed scores for individual cardiovascular events such as stroke or heart failure, as well as for total CVD, reflecting the composite of both fatal and non-fatal cardiovascular events, including scores with and without the use of laboratory measures (9). It is crucial for the user to understand risk scores can differ by endpoint predicted – such as whether revascularization or other soft endpoints are included, if only fatal CVD is predicted, 10-year vs. 30-year or lifetime risk predicted, or whether they are designed for prediction of primary (most scores) or secondary events. For instance, if the user is most interested in evaluating risk of total fatal and non-fatal CVD events the 2008 Framingham risk score equations for primary care published by D'Agostino and colleagues may be most appropriate; these scores predict total CVD and include versions with both total and HDL-cholesterol and without, in which case body mass index is used instead (10).

### 2.2 Other risk score approaches

Given that Framingham risk scores were developed based on a primarily White middle-aged cohort from a small town outside of Boston, Massachusetts, generalizability to other populations has always been a significant concern, and thus other risk scores have been developed globally over the past few decades. Most notably, the European CVD risk scores were originally created for both high and low risk countries in Europe, and in fact have been calibrated for use in most individual countries in Europe (11). This risk score has served as the foundation for risk estimation in the European Society of Cardiology (ESC) Cardiovascular Disease Prevention guidelines (12). They provide cut-points for defining low to very high-risk categories based on age, smoking and levels of blood pressure or LDL-C, from which risk factor goals and treatment approaches are recommended. While the original European Systematic COronary Risk Evaluation (SCORE) algorithm focused on prediction of CVD mortality only, the most recent SCORE2 (13) algorithm now also includes non-fatal CVD events and identifies countries as low, intermediate, high, and very high risk for more precise risk estimation than the prior version of SCORE. As in the original version, diabetes is not included as one of the factors in the algorithm, instead treating diabetes as a high-risk equivalent. More recently, in an attempt to produce a single scoring system that can be used globally, World Health Organization (WHO) risk charts were published to permit risk estimation in 21 global regions (14). These charts may have some limitations as considerable data estimation and modelling were required for many less well-developed regions.

### 2.3 Current US risk score recommendations

**Fig. 1:**
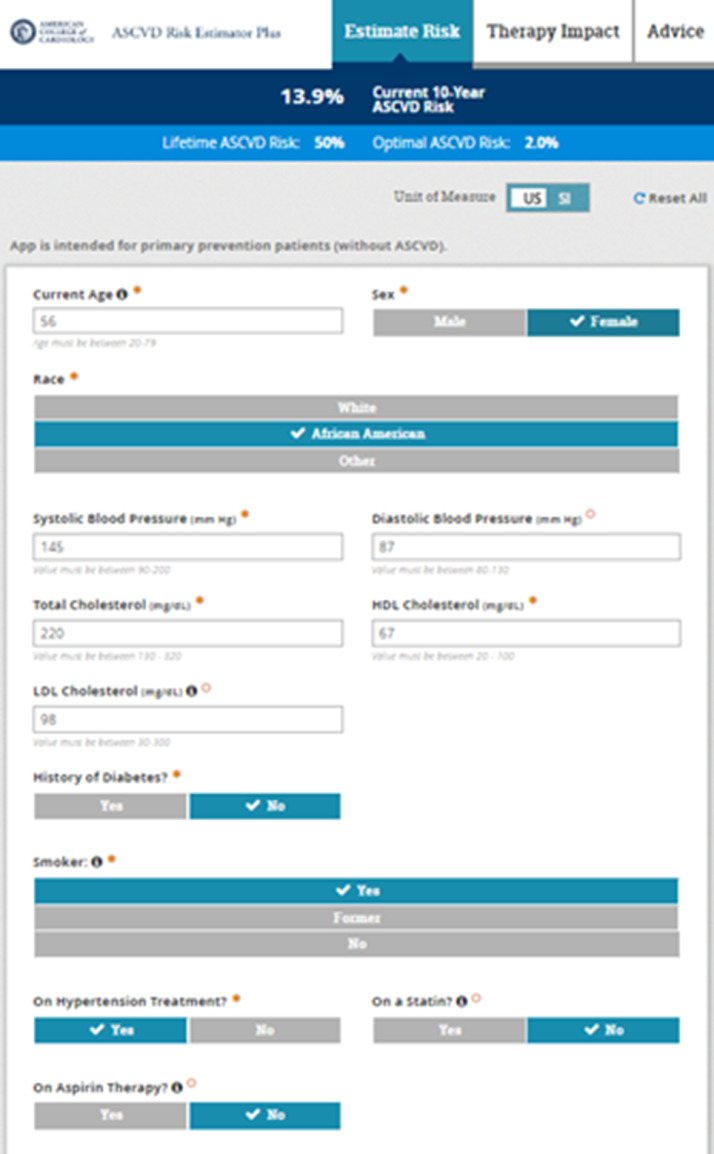
**ASCVD Risk Estimator Plus (Pooled Cohort Equation Risk Score)** tools.acc.org/ascvd-risk-estimator-plus. Provides 10-year ASCVD risk estimates for those aged 40-79 and lifetime ASCVD risk estimates for those aged 20-59.

Both the 2018 Multi-society Cholesterol Management Guidelines (15) as well as the 2019 ACC/American Heart Association (AHA) Primary Prevention of Cardiovascular Disease Guideline (16) recommend the Pooled Cohort Risk Estimator Plus (**tools.acc.org/ascvd-risk-estimator-plus**) (also known as the Pooled Cohort Equations [PCE]) (Fig. 1) for initial CVD risk assessment for those free of known CVD or familial hypercholesterolemia (e.g., those with LDL-C ≥190 mg/dl are assumed to be at high or very high risk). These guidelines note the importance of risk scoring as the initial step in ASCVD risk. Moreover, such risk scoring helps identify higher risk persons where the net clinical benefit is greatest and number needed to treat lowest for preventive treatments such as statin and antihypertensive therapy (17). This scoring algorithm was developed from four major US cohorts consisting of more than 30,000 individuals with at least 10-years of follow-up for CVD events and predicts both 10-year (for those aged 40-79 years) and lifetime (for those 20-59 years of age) risk of ASCVD consisting of fatal and nonfatal CHD and stroke only. Those identified with a 10-year ASCVD risk of ≥20% are given a definite recommendation for statin therapy, whereas those in the 5-<20% range are given consideration for statin therapy depending on the consideration of risk enhancing factors and coronary calcium if needed (discussed further in this document). Moreover, the 2017 ACC/AHA hypertension guideline (18) recommends use of the PCE to guide the use of pharmacologic therapy, which is recommended if the 10-year ASCVD risk is >=10% for those with stage 1 hypertension (130-139 mmHg systolic or 80-89 mmHg diastolic blood pressure) in the absence of diabetes or cardiovascular disease. Estimation of lifetime risk can be very useful, particularly as a motivator to patients to adhere better to lifestyle or pharmacologic therapy, given the fact that many persons at low or intermediate shorter-term risk are at high lifetime risk of ASCVD. Important to note in the PCE and other risk scores focusing on the prediction of hard ASCVD events is the fact that other outcomes such as peripheral arterial disease or heart failure are not included in the estimated risk, thus estimates of total ASCVD events would likely be higher. Moreover, like many other risk scores, it relies on a limited set of risk factors, namely, age, sex, systolic blood pressure, antihypertensive treatment, total and HDL-cholesterol, diabetes, and cigarette smoking, but does provide input to specify Black ethnicity. The 10-year risk of future ASCVD is categorized into those at low (<5%), borderline (5-<7.5%), intermediate (7.5-<20%), and high (≥20%) risk. Importantly, the tool also allows one to set the absence of, or lower levels of a given risk factor to estimate the hypothetical “effect” of reducing or eliminating the risk factor, such as lowering cholesterol levels or stopping smoking. While diabetes mellitus (DM) is included in the PCE, it is treated as a binary factor (as is the case in other risk scores where it is included) and factors such as duration of diabetes and glycated hemoglobin levels are not included. DM is not necessarily a CHD risk equivalent (19,20) warranting the consideration of DM-specific risk scores.

### 2.4 Risk scoring in diabetes

While diabetes risk scores such as those from the United Kingdom Prospective Diabetes Study (UKPDS) (21) have been developed, there is yet no pooled cohort risk score of DM yet developed based on US cohorts. Other risk scores for CVD risk assessment in DM have also been published (22). The ACC/AHA/Multi-society guideline recommends the use of a moderate intensity statin in patients ≥40 years with DM, regardless of 10-year risk estimation. However, the guideline further recommends the use of global risk assessment in those with DM to further risk stratify, with those at higher risk given consideration for high intensity statin therapy (with ezetimibe if needed) to lower LDL-C at least 50%. Various risk enhancing factors, discussed later in this document, such as duration of diabetes or the presence of microvascular complications are considered for informing the treatment decision, particularly in younger persons with DM. While CAC scoring was not recommended by the 2018 guideline for risk stratification in those with DM, its potential role is discussed later in this document in the section on subclinical atherosclerosis.

### 2.5 Other considerations for risk scoring

Current risk scoring approaches focus only on traditional risk factors, which only provide modest discrimination for ASCVD events. Risk scores can underestimate risk in patients from certain racial/ethnic groups, as well as those of lower socioeconomic status or with chronic inflammatory diseases, and overestimate risk in those of greater socioeconomic status or who often utilize preventive healthcare services (17).

There has been consideration for risk scores that incorporate inflammatory measures, social determinants of health, as well as genetic factors. The Reynolds Risk Score was an effort to examine how the additional of the inflammatory measure high sensitivity C-reactive protein (hs-CRP) would add to traditional risk assessment, and found inclusion of hs-CRP was a useful addition to the risk score for women, but not for men (23). Of great interest recently has been the advent of polygenic risk scoring developed from DNA sequencing polymorphisms, which has been shown to strongly predict incident CHD and identify persons with high vs. low genetic risk, also demonstrating a healthy lifestyle to be associated with attenuation of such risk (24). Such scores strongly relate to pre-existing CHD and may also help identify additional persons in primary prevention who could benefit from preventive therapy, such as statins (25). Finally, the consideration of adding social determinants of health (SDOH) was recently demonstrated, where inclusion of 7 SDOH provided excellent discrimination and calibration for identifying prevalent ASCVD (26) However, at present, there are no official recommendations by any major society to incorporate either inflammatory, SDOH, or polygenic risk scoring in clinical practice, but instead these may be considered further for risk assessment (e.g., as risk enhancing factors).

Global risk assessment / scoring therefore forms the foundation for cardiovascular risk assessment, after which the presence of other risk enhancing factors, sex/ethnic-specific considerations and social determinants of health, and screening for subclinical atherosclerosis can further refine risk estimation. This information is integrated to the clinical-patient risk discussion which focuses on a dialogue between the clinician and patient about potential for atherosclerotic cardiovascular disease risk reduction benefits, adverse effects, drug-drug interactions, and patient preferences (27) before the considering initiating or intensifying preventive therapies (**Central Illustration**).

## 3 General risk enhancing factors

In the setting of primary prevention, global cardiovascular risk assessment informs therapeutic decisions regarding initiation or intensification of medical therapies to reduce the risk of ASCVD. Although quantitative risk scoring tools such as the PCE work well at the population level, their performance at the individual level is modest and is often associated with over- or underestimation of ASCVD risk. With that in mind, several risk-enhancing factors were highlighted in the 2018 Guideline on the Management of Blood Cholesterol (15) to refine risk assessment and facilitate shared decision-making. The risk enhancing factors discussed in this section encompass clinical factors and biomarkers.

**Fig. 2:**
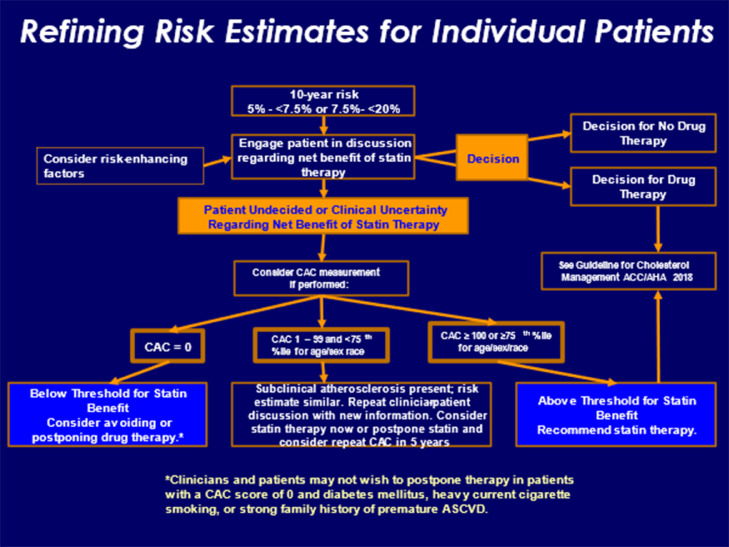
**Refining Risk Estimates for Individual Patients: ASCVD Risk Categories, Risk Enhancing Factors, and Coronary Calcium Scoring.** From Grundy et al. (15).

After quantitative risk assessment is performed using the PCE, the 2018 AHA/ACC cholesterol guideline categorizes individuals into four groups: low risk (<5%), borderline risk (5-<7.5%), intermediate risk (7.5−<20%), and high-risk (≥20%). For all risk groups, a heart healthy lifestyle is recommended. In those considered at low risk, the emphasis is on therapeutic lifestyle changes. On the other hand, in individuals who fall into the high-risk category, initiation of high-intensity statin therapy is recommended in addition to a heart healthy lifestyle (Class I recommendation). Among those at borderline or intermediate risk, evaluation of risk enhancing factors should be pursued to guide decisions regarding statin therapy (Class IIb and I recommendation), respectively. If further guidance on the treatment decision is needed, coronary calcium scoring can be used (discussed further in section below on subclinical atherosclerosis) (Fig. 2). Risk enhancing factors highlighted in the 2018 AHA/ACC cholesterol guideline (15) are detailed in Table 1.

**Table 1:** Risk Enhancing Factors for the Clinician-Patient Discussion.

Adapted from Arnett et a., 2019 (16).
**Family history of premature ASCVD**; (males, age <55 y; females, age <65 y)
**Primary hypercholesterolemia** (LDL-C, 160-189 mg/dL [4.1- 4.8 mmol/L]; non-HDL-C 190-219 mg/dL [4.9-5.6 mmol/L])*
**Metabolic syndrome** (increased waist circumference, elevated triglycerides [>175 mg/dL], elevated blood pressure, elevated glucose, and low HDL-C [<40 mg/dL in men; <50 in women mg/dL] are factors; tally of 3 makes the diagnosis)
**Chronic kidney disease** (eGFR 15-59 mL/min/1.73 m^2^ with or without albuminuria, not treated with dialysis or kidney transplantation)
**Chronic inflammatory conditions** such as psoriasis, RA, or HIV/AIDS
**History of premature menopause (before age 40 y) and history of pregnancy-associated conditions that increase later ASCVD risk such as pre-eclampsia**
**High-risk race/ethnicities** (e.g. South Asian ancestry)
**Lipid/biomarkers**: Associated with increased ASCVD risk
- Persistently elevated, primary hypertriglyceridemia (≥175mg/dL);
- If measured: **Elevated high-sensitivity C-reactive protein** (≥2.0 mg/L **Elevated Lp(a)** A relative indication for its measurement is family history of premature ASCVD. An Lp(a) ≥ 50 mg/dL or ≥125 nmol/L constitutes a risk enhancing factor especially at higher levels of Lp(a) **Elevated apoB** ≥130 mg/dL - A relative indication for its measurement would be triglyceride ≥ 200 mg/dL. A level ≥ 130 mg/dL corresponds to an LDL-C >160 mg/dL and constitutes a risk enhancing factor **ABI (ABI)** <0.9

### 3.1 Clinical factors

Clinical factors associated with increased ASCVD risk include family history of premature ASCVD, primary hypercholesterolemia, metabolic syndrome, sex-specific risk enhancing factors, chronic inflammatory conditions, chronic kidney disease, and high-risk ethnicities. Sex-specific risk enhancing factors and high-risk ethnicities will be discussed in detail in their own sections.

#### 3.1.1 Family history of premature ASCVD

Cardiovascular disease risk assessment has traditionally involved an assessment of family history, particularly of premature ASCVD. Family history provides insight into genetic and environmental exposures. In the 2018 AHA/ACC Cholesterol guideline, a family history of premature ASCVD is defined as a male first degree relative with ASCVD diagnosed before the age of 55 years or a female first degree relative with ASCVD diagnosed before the age of 65 years. A significant proportion of the general population, estimated to be approximately 10-40%, carries a family history of premature ASCVD (28,29). Family history of premature ASCVD is associated with a 1.5-2- fold increased risk of cardiovascular events, but this estimate can vary widely based on the age of the individual at the time of risk assessment, the age of the family member who sustained the ASCVD event, and the number and relatedness of family members (28). It is important to keep in mind that the risk associated with family history is multifactorial, including genetic (increased risk factor burden) and non-genetic influences (adverse lifestyle habits). The importance of a detailed medical history of the number of and which specific first-degree family members are affected with premature CVD (including age and specific CVD sequelae experienced) cannot be overemphasized.

#### 3.1.2 Primary hypercholesterolemia

In the context of the 2018 AHA/ACC Cholesterol guideline, primary hypercholesterolemia is defined as an LDL-cholesterol (LDL-C) of 160–189 mg/dL or a non–HDL-cholesterol (non-HDL-C) of 190–219 mg/dL. The causal relationship between LDL-C and ASCVD is well established based on epidemiologic, experimental, genetic, and randomized placebo-controlled clinical trials (30). Nonetheless, definitions of hypercholesterolemia and treatment thresholds have evolved given results of clinical trials that tested more potent LDL-C lowering therapies. The results of cardiovascular outcome trials with the proprotein convertase subtilisin-kexin type 9 (PCSK9) inhibitors did not identify an LDL-C level below which further LDL-C lowering did not further reduce ASCVD risk (31) While the focus of the 2018 AHA/ACC Cholesterol guideline recommendation has shifted away from absolute LDL-C level at baseline to include more emphasis on statin intensity with a focus on LDL-C reduction within the context of an individual's overall CVD risk, an absolute LDL-C ≥160 mg/dL (and non-HDL-C ≥190 mg/dL) is recognized as a risk enhancing factor given its consistent relationship with ASCVD risk. It should be noted that in those with LDL-C ≥190 mg/dL, global risk scoring is not recommended, and a high intensity statin is indicated in addition to further evaluation for familial hypercholesterolemia (FH).

#### 3.1.3 Metabolic syndrome

The metabolic syndrome is a multifactorial disorder characterized by central obesity, insulin resistance, hypertension, and atherogenic dyslipidemia (particularly high triglycerides and low HDL-C) (32). Given the burgeoning epidemic of obesity, the incidence of metabolic syndrome is on the rise. Current estimates suggest that the prevalence of the metabolic syndrome is 34% among adults under 60 years old and 54% for older adults in the United States (U.S.) (33). The metabolic syndrome includes risk factors that, either individually or collectively, and even in the absence of diabetes, are predictive of poor outcomes and is associated with significant cardiovascular morbidity and mortality (34). Thus, given its relationship to ASCVD, the identification of metabolic syndrome is an important step in risk assessment and ASCVD risk mitigation. Since metabolic syndrome factors such as triglycerides, waist circumference, and glucose levels are not part of most global risk assessment scores such as the PCE, identification of persons with metabolic syndrome is considered a risk enhancer beyond the global risk estimate for further informing the treatment decision. Moreover, the presence of one of its components should prompt a search for the others.

#### 3.1.4 Chronic inflammatory conditions

While the relationship of atherogenic lipoproteins with ASCVD is widely accepted, the contribution of inflammation to atherosclerosis is less commonly appreciated. The 2018 AHA/ACC Cholesterol guideline recognized the increased ASCVD risk among individuals with inflammatory conditions, including rheumatoid arthritis (RA), systemic lupus erythematosus (SLE), psoriasis, and human immunodeficiency virus (HIV) infection. RA is a common inflammatory disease and is associated with a 1.5-2 fold increased risk of ASCVD (35). A meta-analysis including 24 studies and over 111,000 patients found a significant 50% increased risk of cardiovascular mortality among patients with RA (36). SLE is an autoimmune disorder associated with multisystem inflammation. It is associated with a a 2-3 fold higher risk of myocardial infarction (MI) compared to the general population and more profound prematuirty of ASCVD (37). Moreover, the leading cause of mortality amongst those with SLE is CVD (38). Psoriasis is another inflammatory condition, most prominently of the skin. A large meta-analysis including data from over 218,000 patients with psoriasis demonstrated that severe psoriasis was associated with a 70% increased risk of MI, 56% increased risk of stroke, and 39% increased risk of cardiovascular mortality. Mild psoriasis was also associated with significant morbidity — a 29% increased risk of MI and 12% increased risk of stroke (39). Finally, HIV disease, another chronic inflammatory disease, is associated with signifcantly increased risk of CVD. HIV infected individuals have a 2-fold increase risk of CVD when compared to non-infected individuals, even when adjusting for cardiovascular risk factors (40).

#### 3.1.5 Chronic kidney disease

CVD accounts for most of the morbidity and mortality in patients with chronic kidney disease (CKD) (41). CKD increases the risk of CVD-related mortality even after controlling for traditional cardiovascular risk factors such as hypertension and diabetes mellitus. Non-traditional risk factors related to CKD such as anemia and hyperphosphatemia appear to also contribute to CVD risk (42,43). CKD causes CVD through a complex interplay of metabolic alterations, inflammation, oxidative stress, and uremia. Management of the increased risk of CVD in CKD currently focuses on the control of comorbid conditions such as dyslipidemia, hypertension, and diabetes.

### 3.2 Biomarkers

The 2018 AHA/ACC Cholesterol guideline specifically emphasizes a variety of lipid measurements/biomarkers that may be used to refine ASCVD risk estimation. These risk enhancing factors include persistently elevated triglycerides, elevated lipoprotein(a) [Lp(a)], elevated apolipoprotein B (ApoB), elevated high-sensitivity C-reactive protein (hsCRP) (discussed in the next section), and low ankle-brachial index (while classified by this guideline as a risk enhancing factor, it is considered a measure of subclinical disease in other guidelines and discussed in that section of this manuscript). It is important to point out that the guideline does not specifically recommend measuring these biomarkers. However, if these measurements are available and exceed guideline thresholds (see below), they are considered risk enhancing factors.

#### 3.2.1 Triglycerides

The ACC/AHA/Multi-society guideline defines as a risk enhancing factor primary hypertriglyceridemia as persistently (optimally three determinations) elevated triglycerides (≥175 mg/dL). Over the last decade, there has been a renewed interest in the association between elevated triglycerides and ASCVD as randomized controlled trials with HDL-C–raising therapies have failed and genetic studies suggest that triglycerides, not HDL, are causal in the atherosclerotic pathway (44). Moreover, the recent large randomized controlled trial REDUCE-IT demonstrated a significant improvement in cardiovascular outcomes with the use of high dose icosapent ethyl, a potent triglyceride lowering agent, vs. placebo on a background of optimal medical therapy, including statins, in those with known ASCVD or diabetes and multiple risk factors with triglycerides of 135-499 mg/dL (45). This effect was largely independent of the triglyceride-lowering effect, and may be due to other factors such as antioxidant, anti-inflammatory, and cell membrane stabilizing benefits of icosapent ethyl. Several practice guidelines have since recommended the use of this therapy in such patients.

#### 3.2.2 Lipoprotein(a) [Lp(a)]

Lp(a) is an atherogenic particle consisting of a molecule of apolipoprotein(a) (apo(a)) covalently bound to ApoB on the LDL particle (46). The epidemiologic and genetic data consistently demonstrate a significant association between Lp(a) and ASCVD (47,48). The theoretical basis for its atherogenicity relates to both its LDL and apo(a) moieties and to its enriched concentration of oxidized phospholipids. Moreover, given its homology to plasminogen, Lp(a) may interfere with fibrinolysis and thus promote atherothrombosis (49). With regard to ASCVD risk assessment, the measurement of Lp(a) in intermediate risk patients leads to reclassification of 39.6% of individuals into either lower or higher risk categories (50). Thus, the guideline stipulates that a Lp(a) ≥50 mg/dL (or ≥125 nmol/L) constitutes a risk-enhancing factor, especially at higher levels of Lp(a). Clinical trials of investigational therapies for lowering Lp(a) are planned or ongoing to determine their role in ASCVD risk reduction.

#### 3.2.3 Apolipoprotein B [ApoB]

ApoB is a large protein found on the surface of atherogenic lipoproteins and serves as a structural scaffold for lipidation as well as a ligand for the LDL receptor, which facilitates its clearance from the plasma. Since one ApoB is found on each of the hepatically derived atherogenic lipoproteins – very low-density lipoprotein (VLDL), intermediate-density lipoprotein (IDL), LDL and Lp(a), it is an excellent proxy of total atherogenic lipoprotein particle concentration. ApoB performs better than LDL-C when assessing risk of ASCVD (51). The 2018 ACC/AHA cholesterol guideline mentions that ApoB levels may be useful in identifying whether hypertriglyceridemia is associated with increased atherosclerotic risk. There is considerable evidence that ASCVD risk is higher in those with hypertriglyceridemia and high apoB versus those with hypertriglyceridemia and normal apoB levels (52). Thus, when triglycerides are elevated, ApoB can be used as a risk-enhancing factor to determine if a statin should be recommended.

#### 3.2.4 NT-pro-BNP and hs-Cardiac Troponin

These factors were not included as risk enhancing factors in the 2018 AHA/ACC Guideline, however, studies from more than a decade ago showed B-type natriuetic peptide (BNP) levels to strongly predict CVD outcomes. Wang et al. (53) showed in the Framingham Heart Study plasma BNP levels to be associated with first major CVD events, incident heart failure, atrial fibrillation, stroke or transient ischemic attack and death. Moreover, a meta-analysis of 40 long-term prospective studies involving more than 87,000 patients showed the highest versus lowest tertile of BNP levels to be associated with a 2.8-fold greater risk of CVD events, with results similar in the general population compared to those with stable CVD; however, there were only modest improvements in risk discrimination (54). The 2010 ACCF/AHA guideline for assessment of cardiovascular risk in asymptomatic adults, however, did not recommend measurement of natriuretic peptides for CHD risk assessment in asymptomatic adults (55). However, given the strong evidence for naturietic peptides in prognosis in heart failure (56) BNP/NT-proBNP is in the 2017 ACC/AHA Heart Failure guideline for risk stratification in a pre-clinical population to identify those at risk for HF “Stage A/B” for prevention (COR IIa) (before the onset of clinical HF), besides being a measure to guide prognosis and further risk stratification in those with HF (57). It is also in the 2020 ACC/AHA Valvular guidelines for aortic stenosis to guide risk stratification for the timing for intervention (58)

Cardiac troponin levels are well-established in risk assessment for acute coronary syndrome; in particular, the 0-hour/1-hour (0-/1-h) algorithm with high-sensitivity cardiac troponin (hs-cTn) has been recommended for early risk stratification of acute myocardial infarction (class A and evidence level B) (59), besides being recommended to guide prognosis and further risk stratification in those with heart failure, given the many studies showing cardiac troponin to predict prognosis in heart failure (57). Only recently have high-sensitivity methods allowed the accurate detection of cardiac troponin levels in healthy adults and several studies over recent years have shown cardiovascular risk to progressively increase in the general population with hs-troponin levels well below the 99^th^ percentile used in the detection of myocardial injury and/or diagnosis of MI (60). The role of cardiac troponins in cardiovascular risk assessment among asymptomatic populations continues to be an important issue of discussion among many experts (61)

### 3.3 Inflammation

Inflammation is the primary driver of a broad spectrum of disease, including atherosclerosis, diabetes, neurodegenerative disorders, cancer, and autoimmune disorders, to name but a few (62). Virchow identified atherosclerotic disease as a primary manifestation of inflammation over 150 years ago (63). In the modern era of medicine, atherosclerotic disease has become firmly established as an inflammatory disorder on both observational and experimental grounds ([64], [65]). Inflammation is a highly evolved and conserved set of histologic and biochemical responses designed to protect eukaryotes from infection, promote wound healing and eliminate apoptotic/necrotic debris, and provide immunosurveillance to protect against the proliferation of malignant cell lines (66). Chronic inflammation provides the foundation for atherosclerosis by establishing foci along arterial walls characterized by: 1) endothelial dysfunction, 2) increased transmigration of inflammatory white cells into the subendothelial space, 3) a pro-oxidative environment within which apoB-containing lipoproteins can be oxidized and scavenged by activated macrophages, 4) an increase in local prothrombotic tendency, and 5) increased local production of a wide variety of interleukins and cytokines that potentiate cell migration and clonal expansion of different histologic components of the media and adventitia ([67], [68], [69]).

#### 3.3.1 C-reactive protein

During atherogenesis, the levels of specific inflammatory mediators rise. This offers investigators the opportunity to test whether or not they can provide incremental information that can help accurately reclassify risk over and above traditional risk factors such as hypertension, hyperlipidemia, and cigarette smoking. Over the last three decades, hsCRP (a pentraxin and component of the acute phase response) has been the most intensely investigated and validated inflammatory biomarker ([70], [71]) and has been shown to meaningfully reclassify cardiovascular risk estimation ([72], [73]). Elevations in hsCRP are associated with a level of risk on par with elevations in LDL-C. In the Women's Health Study, women with both a high hsCRP and high LDL-C experienced the greatest risk for acute cardiovascular events over 8 years of follow-up, while those with the lowest hsCRP and LDL-C had the lowest risk for cardiovascular events (74). Women with either high LDL-C and low hsCRP or low LDL-C and high hsCRP had risk that was between these two extremes. A number of *post hoc* analyses of secondary prevention statin trials demonstrated that this concept of “dual targets” (which considers residual risk due to inadequate LDL-C reduction and residual inflammatory risk with specific cut points for LDL-C and hsCRP) was reproducible and significant. In the Pravastatin or Atorvastatin Evaluation and Infection Therapy trial (PROVE-IT)(75), Aggrastat to Zocor trial (A–Z) (76), and IMProved Reduction of Outcomes: Vytorin Efficacy International Trial (IMPROVE-IT)(77) the patients with the lowest on-trial rates of CVD events were those with the lowest LDL-C and hsCRP; those with the highest rates had the highest levels of these two biomarkers. Similarly, in the Air Force/Texas Coronary Atherosclerosis Prevention Study (AFCAPS/TexCAPS) and Justification for the Use of Statins in Prevention: An Intervention Trial Evaluating Rosuvastatin (JUPITER)(78) both primary prevention trials, the validity of dual targets was confirmed. The therapeutic reduction of a biomarker reflecting systemic inflammatory status with a statin provides incremental cardiovascular risk reduction over and above LDL-C lowering. Hence, residual risk is partly comprised of risk directly attributable to inflammation.

#### 3.3.2 Other inflammatory biomarkers

Several biomarkers have been of interest to many investigators; however, do not have any current recommendations for their routine measurement or clinical use. While not included as risk enhancing factors in the 2018 ACC/AHA/Multisociety guidelines, they may be helpful to the clinician in identifying those at increased ASCVD risk warranting greater efforts for risk reduction using existing therapies.

*Lipoprotein-associated phospholipase A2 (Lp-PLA_2_)* is an enzyme that associates with serum lipoproteins. Lp-PLA_2_ hydrolyzes phospholipids and produces bioactive, pro-inflammatory lipids ([79], [80], [81]) and is an inflammatory marker specific to atherosclerotic disease. Its validity as an independent risk factor for ASCVD has been demonstrated from multiple longitudinal studies ([82], [83]). However, a major clinical trial of daraplabib failed to show lowering Lp-PLA2 to reduce risk of CVD events (84).

*Myeloperoxidase (MPO)* is a heme peroxidase secreted by neutrophils, monocytes, and macrophages (85). MPO chemically modifies HDL particles, thereby rendering them dysfunctional and no longer capable of engaging in reverse cholesterol transport (86). Serum myeloperoxidase levels correlate with risk for ASCVD related events ([87], [88])

*Myeloid-Related protein 8/14* is a heterodimeric protein complex and is a member of the alarmin family that activates a variety of inflammasomes (89), vascular inflammation, leukocyte activation and migration, thrombosis, stimulates tissue factor (a procoagulant) production, and is increased in acute MI ([90], [91])and within atherosclerotic plaques (92). The risk of a recurrent cardiovascular events is increased with increasing quartiles of MRP-8/14 ([93], [94]).

*Matrix Metalloproteinases (MMPs, also known as matrixins)* comprise a large family of serine endopeptidases and can be injurious and pro-inflammatory, thining and weakening the fibrous cap making it more prone to rupture ([95], [96], [97], [98]).

*Lectin-Like Oxidized Low-Density Lipoprotein Receptor-1 (LOX-1)* is highly expressed by endothelial cells, smooth muscle cells, and platelets (99). LOX-1 is an oxidized LDL receptor and induces endothelial dysfunction, oxidized LDL scavenging, apoptosis, smooth muscle migration, as well as platelet activation/aggregation (100). Serum levels of LOX-1 increase as a function of plaque severity, the number of coronary arteries affected, levels of tumor necrosis factor-α (101) and also correlate with the number of complex coronary lesions (102).

*Growth Differentiation Factor-15 (GDF-15; aka macrophage inhibitory factor-1)* is highly expressed in the settings of heightened inflammation and myocardial ischemia (103), is a regulatory switch for inflammation, cellular apoptosis, and angiogenesis (104), and predicts all-cause mortality and CVD (105).

#### 3.3.3 Recent clinical trials testing the inflammation hypothesis

More recent clinical trials confirm the importance of reducing the intensity of inflammation in patients with ASCVD. The Canakinumab Anti-inflammatory Thrombosis Outcome Study (CANTOS) randomized 10,060 patients with a prior history of MI and an hsCRP ≥2.0 mg/L to either canakinumab (a monoclonal antibody directed against interleukin-1β (IL-1β)) or placebo (106). IL-1β is produced via activation of the NLRP3 inflammasome and is a potent trigger of inflammation ([107], [108]). The 150 mg dose of canakinumab reduced the primary (nonfatal MI, any nonfatal stroke, or cardiovascular death in a time to-event analysis) and secondary (included the components of the primary end point as well as hospitalization for unstable angina that led to urgent revascularization) end points in a statistically significant manner by 15% and 17%, respectively. Benefit was independent of changes in lipids (lipid levels did not change). Serum levels of hsCRP decreased with canakinumab therapy. A subgroup analysis of the CANTOS trial showed that for patients with hsCRP <2.0 mg/L treated with canakinumab, cardiovascular mortality and all-cause mortality were both reduced by 31% compared to the group whose hsCRP ≥2.0 mg/L. Colchicine exerts anti-inflammatory effects and is used to treat gout and pericarditis. The Colchicine Cardiovascular Outcomes Trial (COLCOT) randomized 4745 patients who had sustained an MI within 30 days to treatment with either low-dose colchicine or placebo (109). Median follow-up was 22.6 months. The primary (CV mortality, resuscitated cardiac arrest, MI, stroke, or urgent hospitalization for angina leading to coronary revascularization) and secondary (CV mortality, all-cause mortality, MI, stroke, and resuscitated cardiac arrest) end points were significantly reduced by 23% and 15%. The CANTOS and COLCOT trials provide proof of concept that attenuating inflammation reduces risk for acute cardiovascular events. There are, however, no current national or international recommendations for the use of either canakinumab or colchicine for the targeting of inflammation for reduction of ASCVD risk.

## 4 Female-specific risk enhancing factors and risk stratification considerations in women

### 4.1 Epidemiology of CVD risk in women

CVD is the leading cause of death of women in the U.S. and worldwide (110). Approximately 44% of U.S. women age ≥20 years (n=60,800,000) are living with prevalent CVD. Despite initial declines in CVD mortality in women after the year 1999 following the launch of the first women-specific prevention guidelines (111), more recent data have shown a stagnation in this progress, with even a slight uptick in CVD mortality since 2015 (110). In fact, heart disease mortality rates have been accelerating the fastest among middle aged women ([112], [113]). Therefore, it is of upmost importance that we improve upon CVD risk assessment and implementation of lifestyle and pharmacologic preventive strategies. Unfortunately, a recent AHA survey has indicated that awareness of heart disease being the leading cause of death in women has declined over time (114). This reduced awareness was particularly noted among younger women, who might benefit the most from primordial and primary prevention, and also among racially/ethnically underrepresented women who shoulder an increased burden of social and health inequities (115).

### 4.2 Pooled cohort equations and other risk estimation tools in women

Global risk tools such as the PCE are race- and sex-specific and predict hard ASCVD including CHD and stroke. However, studies have found that the PCE (or other similar risk estimation equations) both overestimate risk, such as in populations at higher socioeconomic status (116), or underestimate risk, such as in populations with more social deprivation (117) or among individuals with risk factors not captured in the PCE such HIV, auto-immune disease, CKD, or family history of premature CHD ([118], [119], [120]). Moreover, in a meta-analysis examining the performance of the PCE and other risk equations for predicting 10-year risk of ASCVD in women compared to men found that the “observed to expected” ratio for the PCE was 0.76 [95% CI: 0.65, 0.88). In other words, the number of observed events were fewer than that predicted by the equations (i.e., the PCE tends to over-estimate risk in women) (121).

*Sex differences in traditional risk factors.* There can be disparity in risk conferred by even traditional CVD risk factors. Certain traditional risk factors, such as smoking and diabetes, confer relatively greater risks of CVD in women compared to men ([122], [123]). One systematic review, including 64 cohort studies, found that diabetes in women conferred a 40% greater risk of incident CHD compared to diabetes in men (123).

### 4.3 Risk enhancing factors in women

#### 4.3.1 Auto-immune disease

Auto-immune diseases affect approximately 8% of the population and are more prevalent in women (∼80%) (124). As discussed above, autoimmune diseases, such as RA and SLE, are associated with increased CVD risk beyond the burden of traditional CV risk factors ([125], [126], [127], [128], [129]) and increased prevalence of premature atherosclerosis ([130], [131]). As such, they are considered “risk-enhancing” factors in the 2019 ACC/AHA Primary Prevention Guideline (16). In addition to the disparity conferred by traditional risk factors and the excess female burden of autoimmune disease, women also experience unique risk factors throughout their lifetime related to pregnancy, hormones, and menopause that men do not experience ([132], [133]).

#### 4.3.2 Menarche

Early menarche (i.e., the onset of menses) before age 10-11 years has been associated with a ∼10-25% increased risk of CVD across population studies ([134], [135], [136]). Late menarche after the age of 17 has been also associated with increased CVD risk. Although a history of early menarche is associated with a worse cardiometabolic profile, including a higher body mass index (BMI) at middle-age, the association of early menarche and future CVD risk remained significant even after adjusting for adiposity (136).

#### 4.3.3 Polycystic ovary syndrome (PCOS)

PCOS, particularly the hyperandrogenism subtype, is associated with an adverse cardiometabolic profile in women, hallmarked by elevated BMI, dyslipidemia, and elevated blood pressure, compared to similarly aged women without PCOS (137). Insulin resistance is a central feature of PCOS. PCOS has been associated with a 2-fold increased risk of future CVD (138), and while the greater prevalence of CVD risk factors may explain this risk in part, the excess risk does not appear to be entirely explained by the elevated BMI (139).

#### 4.3.4 Oral contraceptives

Oral contraceptive use with estrogens, alone or combined, has been associated with increased risk of stroke; in contrast, this is not the case with progestin-only contraceptives (135). Oral contraceptive use combined with smoking synergistically increases a woman's risk for CVD and venous thromboembolism (VTE) ([140], [141]).

#### 4.3.5 Infertility

There have been conflicting reports whether infertility is an independent CVD risk factor in women or not. The Study of Women's Health Across the Nations (SWAN) did not find infertility to be an independent risk factor for CVD events (142), although other studies have. The discrepancy may be due to the underlying causes of infertility, whether there was use of assisted reproductive technology, or whether studies followed women for a sufficiently long enough time for CVD events to accrue. CVD risks associated with infertility may be associated with the underlying risk conditions of PCOS and premature ovarian insufficiency (POI). Fertility treatments may be associated with increased risks of gestational hypertension (143) and stroke (144), and the CVD risks appear to be greatest among women with unsuccessful (failed) fertility treatment, suggesting these women should be monitored long-term for CVD risk (145).

#### 4.3.6 Parity and breast feeding

Several studies have linked grand multiparity (≥4 or 5 live births) with increased risk of CVD ([146], [147], [148]), whereas breastfeeding is associated with lower risk (146). The mechanisms are not entirely clear in women with multiple live births but may be mediated through weight gain, dysregulation of adipokines, and increased inflammation ([149], [150]).

#### 4.3.7 Adverse pregnancy outcomes (APOs)

Maternal mortality is on the rise in the U.S. and the leading cause of maternal morbidity and mortality is from CVD, which disproportionately affects Black women (151). Black women are about 50% more likely to develop pre-eclampsia compared to White women (152).

#### 4.3.8 Preeclampsia

Preeclampsia is associated with increased risk of acute CV complications around the time of delivery, including peripartum cardiomyopathy (152). But even beyond the acute period post-delivery, there has been increasing recognition that APOs such as hypertensive disorders of pregnancy including pre-eclampsia, gestational diabetes, preterm delivery, and having a small for gestational age infant are independently associated with long-term maternal CVD risk, with events sometimes occurring more than a decade after the index pregnancy ([153], [154]). Systematic reviews have found that preeclampsia was associated with a 2-fold increased risk of future CHD, stroke, or CV death and a 4-fold increased risk of heart failure, even after adjustment for potential confounding factors (135,155). Gestational hypertension also appears to be associated with elevated CVD risk (135). *Preterm delivery*, which often accompanies preeclampsia, was also associated with be associated with a 2-fold increased risk of CVD (155,156).

#### 4.3.9 Gestational diabetes mellitus (GDM)

GDM is strongly associated with the development of type 2 diabetes (T2D) within 10 years of index pregnancy (157), and thus women should be followed closely for their glucose status. Further, GDM is associated with an approximately 2-fold increased risk of CVD risk (135,138,158,159); some of that risk is due to the development of T2D, but women with a history of GDM who do not develop T2D still seem to have a 50% increased risk of CVD compared to women without GDM (159).

#### 4.3.10 Early menopause

The average age of natural menopause of U.S. women is around age 52 years. Early menopause (both natural and surgical) before the age of 45 has been shown to be associated with increased risk of incident CVD by about 30-50%, even after accounting for other traditional risk factors (134,135,160,161).

#### 4.3.11 Premature ovarian insufficiency (POI)

POI, a more extreme form of early menopause, is a loss of normal function of the ovaries before the age of 40 and occurs in 1% of women (162). POI has also been similarly shown to be associated with an 60-70% increased risk of future CHD and CVD (135,162), as well as an increased risk for mortality (163).

#### 4.3.12 Menopause

At time of menopause, women experience unique changes in CV risk profile related to the cessation of endogenous estradiol production including a rise in total cholesterol and LDL-C and increased deposition of visceral adipose tissue (134). The post-menopausal ovary continues to secrete testosterone. Women with a more androgenic (“male-like”) sex hormone pattern after menopause are shown to have increased risks for coronary artery calcification (CAC), CVD, and heart failure (164,165).

#### 4.3.13 Vasomotor symptoms (VMS)

VMS (i.e., “hot flashes”) are known for their adverse effects on quality of life for women in the peri-menopausal and immediate post-menopausal period; yet emerging evidence suggests menopausal symptoms may also be a risk factor for CVD (135). Women with more frequent VMS or who experience VMS for longer period of times after the final menstrual period also have increased risk for incident CVD compared to women who do not (166), suggesting that frequent and persistent VMS may be a novel risk factor for CVD.

### 4.4 Considerations for lifetime risk assessment in women

An important issue for risk assessment in women is that women are often to be estimated to be at low or borderline risk in the short-term over the next 10-years but have high risk over their lifetime. Statistics, including from the US and Europe often show age-specific CVD risks to be lower in women compared to men; however, risks in women lag men by approximately 10 years. Data from the National Health and Nutrition Examination Survey (NHANES) from 2003 to 2006, estimated that 61% (n=47,400,000) of U.S. women have low short-term but high lifetime predicted risk (167). However, prevention efforts are more effective when implemented at a young age. The presence of a single major risk factor by middle age is associated with increased CVD risk and reduced longevity. For example, at index age of 45 years, women with all risk factors optimal lived up to 14 years longer free of total CVD than women with at least 2 risk factors (168). Therefore, a focus on only short-term 10-year risk may lead to failure to implement preventive strategies in women.

The incidence of stroke in young women aged 25 to 44 years is higher than in similar aged men (169), which may be due to sex-specific factors related to pregnancy, preeclampsia, oral contraceptive use, and conditions such as migraines (170) and auto-immune disease. Again, this age range is predominantly outside of the range that the PCE is applied to, and these factors are not included in the PCE.

### 4.5 Incorporating sex-specific factors into risk assessment

Although preeclampsia has been shown to be independently associated with CVD risk, studies that have attempted to include APOs into risk scoring equations that include traditional CVD risk factors only found that approach led to small improvements in discrimination and net reclassification (171). This may be because these studies used population-based cohorts that primarily included women beyond their reproductive years, and future studies should include study samples that are closer to the target population of women intended for CVD screening and preventive interventions following an adverse pregnancy outcome (133).

### 4.6 Subclinical atherosclerosis in women

CAC, indicative of calcified coronary plaque and a surrogate marker of total coronary atherosclerotic burden, has emerged as a superior predictor of risk in women, above age and other traditional risk factors (172,173). The presence of CAC (score >0) provides incremental risk prediction even among women considered to be a low risk by traditional risk scores such as the PCE (174,175). Although at a given age, women are less likely to have prevalent CAC compared to their men counterparts, when CAC is present, it confers a greater relative risk of CVD and mortality in women compared to men (176,177).

## 5 Racial/ethnic specific risk enhancing factors and social determinants of health

Although ASCVD risk assessment is used to guide pharmacotherapy and shared decision making, especially in middle aged and older adults, there may be inaccuracies when applied across diverse racial/ethnic populations. Based on self-identified status, contemporary race/ethnic categories should use these terms as adjectives (e.g., Black people, White people, etc.) instead of nouns (e.g., *Blacks, Whites*, etc.) (178,179). However, race/ethnicity are probably social constructs rather than reflecting true biologic or genetic differences and a higher ASCVD risk burden is usually driven by multiple social determinants of health (SDOH), impacting the probability of future MI, ischemic stroke, and cardiovascular death.

### 5.1 Race/ethnicity considerations in ASCVD risk score algorithms

The ASCVD Risk Estimator or PCE is the most widely used U.S. It includes age, race, sex, systolic blood pressure, treatment for hypertension, total cholesterol level, HDL-C level, DM status, and smoking status ([15], [16]). Non-Hispanic Black (NHB) or African American individuals have increased ASCVD risk compared to non-Hispanic White (NHW) individuals, and higher morbidity and mortality, including CHD and stroke. Overall, NHB adults are two to three times more likely to die from CVD than NHW adults, leading to reduced life expectancy and unacceptable health inequities (180,181). Optimally, risk assessment should include Hispanic/Latinx adults and various categories of Asian American and Pacific Islander adults, including Native Hawaiian, and American Indian/Alaska Native (AI/AN) individuals.

The earliest ASCVD risk models were developed based on Framingham Heart Study data, including the Original, Offspring and Third Generation cohorts, who were predominantly of European descent (182,183). The FHS cohort has been supplemented to include racial/ethnic minority groups reflecting evolving demographic characteristics in the greater Framingham area. Omni-1 and Omni-2 cohorts include individuals of African American, Hispanic/Latinx, Asian, Indian, Native American and Pacific Islander descent (184,185). The degree to which ASCVD risk prediction varies versus actual ASCVD events remains difficult to verify, but the link in recent years between measured lipids and CVD events presently may be less accurate, impacted to some extent by the widespread use of statins (186).

Subsequently, the Jackson Heart Study noted that the 2013 PCE was acceptable as a clinical tool to predict ASCVD in Black patients (187). But the PCE may not accurately estimate risk in Hispanic/Latinx adults, Asian American, including South Asian adults and AI/AN adults, indicating the need for improved models (188). Hispanic/Latinx individuals do not have increased risk in the current PCE, but this subpopulation is very heterogeneous and future consideration of ASCVD risk should disaggregate Hispanic/Latinx groups (188). Furthermore, one study showed risk overestimation of 30-40% in Hispanic/Latinx populations when the non-Hispanic White PCE was tested in Hispanic/Latinx participants (189).

A significant limitation of the present 2018 ACC/AHA Cholesterol guideline (including both the PCE as well as treatment-related considerations) is the lack of recognition of disadvantaged socioeconomic status (SES), which has been consistently associated with higher ASCVD risk and also utilized in British ASCVD risk estimates ([190], [191], [192], [193]). The current PCE also does not include the potent ASCVD risk factor, Lp(a), which appears to provide additional risk assessment, especially in NHB and South Asian adults (4, 194), although it is used in the 2018 guideline as a risk enhancing factor. This addition of Lp(a) to risk assessment for South Asians is in addition to the other factors that determine SA ancestry as a “ASCVD risk enhancer”, including visceral adiposity and insulin resistance, despite comparatively low BMI (195). Among 12,149 ARIC (Atherosclerosis Risk In Communities) participants, 23% of whom were NHB adults, family history (FHx) and elevated Lp(a) were independently associated with ASCVD and may be useful concurrently for guiding primary prevention therapy decisions (194).

### 5.2 Other ASCVD risk scores for use in diverse populations

Beyond the original Framingham Risk score and the present AHA/ACC risk calculator, although not based on American cohorts, United Kingdom (UK) risk calculators may be informative of racial/ethnic populations, including African descent and South Asian individuals (16, 196,197). These include the UK QRISK2 (197) which estimates the 10-year risk of MI or stroke and includes South Asian ethnicity as an additional risk factor as well as the ETHRISK score (198) which is a recalibration of Framingham risk score in seven British Black and minority racial/ethnic groups. Additionally, the Multi-Ethnic Study of Atherosclerosis (MESA) (N=6814) includes racial/ethnic diversity: 38% White, 28% Black, 23% Hispanic, and 11% Chinese, including 50% women. The MESA CHD Risk Score was first to consider CAC in risk estimation. Moreover, MESA demonstrated how risk factors led to subclinical disease and associated events (199).

### 5.3 Race/ethnicity considerations in ASCVD risk assessment

In non-Hispanic Black (NHB) adults, hypertension has a more substantial impact in NHB adults, but DM demonstrates a less significant effect on ASCVD risk among NHB adults compared to NHW and Hispanic/Latinx men. Although not a component of the present ASCVD risk calculator, left ventricular hypertrophy (LVH), especially in NHB adults, may reflect markedly higher ASCVD risk and is tied to hypertension, older age, and obesity. Nevertheless, ECG LVH may not add significantly to risk based on traditional cardiovascular risk factors (200). Future research is needed to determine whether modern tools including echocardiography and magnetic resonance imaging will add to the LVH risk assessment value in NHB and others (201). In addition, CAC in multiple racial/ethnic groups appears useful in reclassifying risk, especially with intermediate risk ([202], [203]).

Asian American subgroups are quite heterogenous and effort should be taken to recognize differences within this population, specifically, various persons of Asian descent, including Asian Indian, Filipino, Japanese, and Vietnamese populations (204,205). Although, in general, Asian American adults are listed to have lower ASCVD risk than NHW adults there is a higher prevalence of LDL-C among Asian Indian, Filipino, Japanese, and Vietnamese populations than among NHW adults (196). Moreover, ASCVD risk in South Asians may be underestimated with the present tool (196).

There are limited ASCVD risk data for indigenous U.S populations, including American Indian (AI)/Alaska Native (AN) individuals. However, AI/AN have high rates of ASCVD risk factors compared to NHW people, including obesity and diabetes with an average life expectancy reduced by 5.2 years compared to the general U.S. population (206,207). Risk in AI/AN also are affected by higher prevalence of non-lipid risk factors (hypertension, diabetes, physical inactivity and low SES) (31).

### 5.4 Social Determinants of Health (SDOH)

Not well-integrated into most prevention guidelines, SDOH can have important further implications in modifying a person's ASCVD risk. They include but are not limited to factors such as unemployment, lack of health insurance or inability to pay medical bills, low income and other measures of economic stability, psychological distress, delayed care due to lack of transport, food insecurity, downward social mobility and educational attainment. A polysocial risk score, such as developed by Javed et al. can help to further inform a person's “social risk” (26). Information on SDOH should always be considered in a clinician's further assessment of one's risk of ASCVD and other health conditions and in assessing a patient's possible access and adherence to lifestyle and medical therapies.

## 6 Role of subclinical atherosclerosis in cardiovascular risk assessment

### 6.1 Rationale and criteria for measuring subclinical atherosclerosis

While global risk scoring is recommended by most guidelines as the first step in CVD risk assessment, it is well-established that persons experiencing CVD events may have few traditional risk factors (208), and thus risk scoring has significant limitations in identifying those who will actually sustain an ASCVD event. Moreover, while the consideration of risk enhancing factors can further individualize risk assessment, many experts support assessing atherosclerotic burden to best predict future ASCVD events. Such tests for measuring atherosclerosis should fit key criteria including: 1) detecting the disease of interest with adequate sensitivity and specificity, 2) being sufficiently reproducible, 3) detecting those where early intervention can be beneficial, 4) providing predictive value over office-based risk assessment (209), 5) having equitable access, and 6) demonstrating cost-effectiveness. It should be understood that imaging strategies to detect subclinical ASCVD are diagnostic tests, not risk factors as such, but techniques by which detecting the presence of atherosclerosis in an asymptomatic person indicates increased risk– in the continuum of risk, it may be thought of as bridge between the conventional concepts of primary and secondary prevention.

### 6.2 Major tests for measuring subclinical atherosclerosis

Multiple tests have been proposed and used in clinical practice and long-term observational studies to determine the ability to measure subclinical atherosclerosis and prediction of ASCVD risk. The parameters most often evaluated to determine the robustness of these tests are net reclassification improvement (NRI), c-statistic from the receiver operator curve, calibration and discrimination.

#### 6.2.1 Ankle-Brachial Index (ABI)

Ankle-Brachial Index (ABI) is a test that is easy, non-invasive, can be done in any office setting without additional equipment by ascertaining the blood pressure of both arms and both legs. The ABI is the ratio of the systolic blood pressure at the ankle (measuring the pressure just proximal to the dorsalis pedis or posterior tibial artery) and compared to the systolic blood pressure at the brachial artery (standard arm assessment) in both arms and legs. A value less than 0.9 is indicative of peripheral artery disease (PAD) (210). A ratio is obtained, normally >1.0, as there is normal potentiation of the blood pressure as it transmits down the aorta. Used primarily for diagnosis of PAD, it has also been studied as a marker of ASCVD risk and atherosclerosis in the peripheral arteries. Several large epidemiologic studies failed to demonstrate robust prediction of abnormal ABI for ASCVD. Multiple cohort studies revealed that traditional risk factors were equally applicable as predictors of incidence of PAD, and ABI was not an independent predictor of CHD ([211], [212]). The Ankle-Brachial Index Collaboration reported a meta-analysis of 16 studies in 2008. While the FRS shows modest discrimination, with a C-statistic for CVD events of 0.646 (95% CI, 0.643–0.657) in men and 0.605 (0.590–0.619) in women (213), several studies show slight improvement of the C-statistic for ABI and very modest reclassification (214). In MESA, ABI had very modest effects and only CAC had robust reclassification and improvement in the c-statistic (215). However, it has been recommended in the 2018 AHA/ACC Guideline on Management of Blood Cholesterol for additional risk assessment in patients with borderline or intermediate risk. However, upon further review by the US Preventive Services Task Force (USPSTF), they concluded that the current evidence is insufficient to assess the balance of benefits and harms of adding the ABI in asymptomatic persons for purposes of screening. The USPSTF found adequate evidence that adding the ABI, hsCRP, and CAC score to existing CVD risk assessment models (i.e., FRS or PCE) may improve calibration, discrimination, and reclassification, however The USPSTF found inadequate evidence to assess whether treatment decisions guided by ABI, hsCRP, or CAC score test results, in addition to existing CVD risk assessment models, lead to reduced ASCVD events or mortality. It remains most often used in clinical practice as a predictor of obstructive PAD in patients with possible claudication and may thus be most useful in older persons with multiple risk factors who have a greater likelihood of PAD. For asymptomatic persons, an ABI <0.9 is considered to indicate increased risk of CVD events (as a risk enhancing factor in the ACC/AHA 2018 guideline), thereby warranting initiation or intensification of risk factor management.

#### 6.2.2 Carotid IMT/Plaque assessment

Because atherosclerosis is generally widespread in those afflected, it is logical to examine whether detecting asymptomatic vascular disease detected by ultrasound scanning of the carotid arteries may refine risk estimation. Carotid ultrasound can provide information on both intimal-media thickness (IMT) and carotid plaque imaging. In general, damage is defined as the presence of IMT >0.9 mm or established plaque. IMT can be non-invasively determined from ultrasound of the carotid arteries and has been used for decades in clinical trials and risk assessment. IMT is a combination of intimal (atherosclerosis) and medial (vascular hypertrophy) changes of the various vascular territories. Multiple trials have used serial IMT for assessment of changes of carotid wall thickness, with reduction deemed to be a positive change (toward normal blood vessel), and increases representing worsening hypertrophy and/or atherosclerosis.

However, lack of standardization and poor reproducibility of ultrasound imaging techniques outside of research laboratories became a major challenge and led to less clinical use and reduced enthusiasm for this tool to help risk stratify asymptomatic persons. Lack of consensus over specific methods to evaluate IMT (far wall vs near wall of carotid, internal vs common carotid, full segment versus predetermined length) has resulted in poor standardization of assessment methods between laboratories or studies. Given these and other conditions, the ACCF/AHA guidelines (216) deemed this measure Class III (no benefit), largely due to suboptimal performance measures on reclassification and risk prediction, along with strong concerns related to reproducibility of the measurement outside the clinical research laboratory. The recommendation stated “CIMT is not recommended for routine measurement in clinical practice for risk assessment for a first ASCVD event”.

Carotid plaque imaging, also available on the same ultrasound probe gave rise to more enthusiasm, as a visible plaque in the carotid artery was seen as direct evidence of atherosclerosis, with stronger data on risk outcomes and reclassification. In MESA, CAC proved improved prediction of CVD and CHD more than carotid plaque (217). Mean IMT≥75th percentile (for age, sex, and race) did not predict events in this cohort. CAC and carotid ultrasound plaque imaging performed similarly for stroke/transient ischemic attack event prediction in the MESA cohort. However, the combination of CIMT with plaque does appear to significantly improve the c-statistic or NRI (218) and guidelines from the ESC in 2021 do provide a Class II LOE B recommendation for carotid plaque assessment as an alternative when CAC scoring is not available (219). There has also been greater interest in combined imaging of plaque both the femoral and carotid arteries, which may offer further improvement in assessment of ASCVD risk (220).

#### 6.2.3 Endothelial function

Assessment of endothelial function is another approach that has not been widely adopted in clinical practice or guidelines for assessment of ASCVD risk. There are many methods used, including brachial artery reactivity, large vessel (aorta) or small vessel assessment of vascular health that have been validated as measures of arterial compliance and have been used clinically. Reproducibility generally is considered good. Many studies demonstrate that these non-invasive methods, such as digital thermal monitoring, strongly correlates with the presence and extent of coronary artery disease (221). However, these modalities have less influence on net reclassification, and less predictive power in large epidemiologic studies than other tests (217). Given the non-invasive nature, the small footprint and amenability to office-based testing, these tests deserve further study to define their independent role, if any, in risk estimation.

#### 6.2.4 Coronary artery calcium testing

**Fig. 3:**
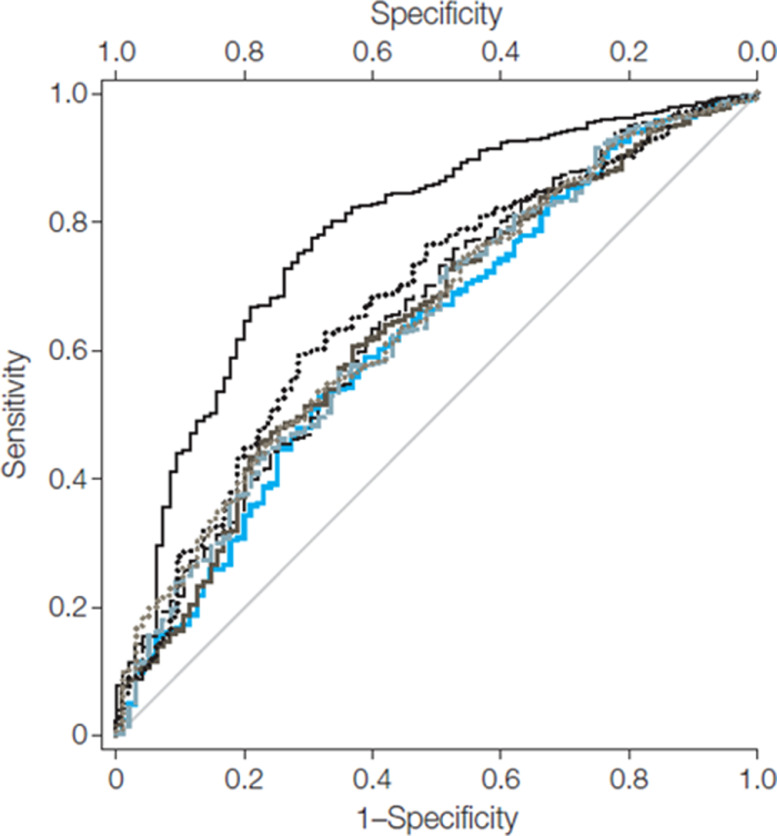
**Comparison of Novel Risk Markers for Improvement in Cardiovascular Risk Assessment in Intermediate Risk Individuals.** From Yeboah et al. (210). Intermediate Risk MESA Subjects (n=1330) C-statistics: FRS alone 0.623; FRS+CAC 0.784 (p<0.001); FRS+CIMT 0.652 (p=0.01); FRS+FMD 0.639 (p=0.06); FRS+CRP 0.640 (p=0.03); FRS+FamHx 0.675 (p=0.001); FRS+ABI 0.650 (p=0.01)

CAC is widely available, extensively studied, and a highly specific measure of subclinical atherosclerosis (222). It is an excellent predictor of ASCVD and predicts both stroke and CHD (223). CAC testing facilitates the enhancing or de-risking of asymptomatic patients and provides a model for initiating or intensifying preventative therapies, including blood pressure and cholesterol treatment as well as aspirin initiation. Currently the most robust method to detect subclinical atherosclerosis, with the strongest outcome data (NRI and improvement in c-statistic) is CAC testing (Fig. 3) (215). This test has shown powerful risk assessment, independent and incremental to traditional risk factors in dozens of large studies. It has been embraced by numerous guidelines and scientific statements, and recently incorporated into the ACCF/AHA Cholesterol Guidelines and Preventive Guidelines to improve risk prediction and guide treatment for high blood pressure, statin use, counseling on healthful diet and physical activity (15). In the guideline it is noted that unless the patient has diabetes, strong premature family history of ASCVD, or is a heavy cigarette smoker that a 0 calcium score can warrant withholding or delaying statin therapy, whereas a positive calcium score under 100 or below the 75^th^ percentile for age, sex, and race can be an indication to consider a statin, and a calcium score of ≥100 or >75^th^ percentile is a definite indication for statin use (Fig. 2). Further support for not withholding statin use in those with diabetes or cigarette smoking derives from more recent follow-up data from MESA indicating many (but not all) such persons with 0 CAC to have 10-year ASCVD risk above the 7.5% net clinical benefit threshold (224). In addition, while the USPSTF has recently downgraded their recommendations for the use of low-dose aspirin use in primary prevention due to minimal net clinical benefit (225), the 2019 ACC/AHA primary prevention guideline notes that CAC screening may identify those at higher risk where aspirin use may be favorable for adults at low risk of bleeding (16). Observational data support the use of low dose aspirin in persons with CAC > 100 (226). Uniting CAC risk stratification with lipid lowering and aspirin treatment individualizes primary ASCVD prevention and shared clinician-patient decision making (227). CAC testing also further improves risk prediction in persons with diabetes beyond traditional risk factors (228); such information may be useful in decisions to consider further intensification of preventive therapy (e.g., statins).

#### 6.2.5 Cardiac Computed Tomography angiography (CCTA)

CCTA, as a standard of care for diagnosis of obstructive CAD in symptomatic persons, is emerging as an alternative in selected cases for screening/assessment of asymptomatic persons (229). The ability to visualize non-calcified plaque, stenosis and cardiac abnormalities (shunts, clots, tumors, anomalous vessels and congenital heart disease), in addition to underlying atherosclerosis, makes this an attractive test to fully visualize the heart health of an asymptomatic person at risk of ASCVD. The radiation doses continue to drop and now are approaching that of a calcium scan (<1 mSev) in many cases. However, the increased cost, requirement for contrast and processing/reading times still preclude it from becoming a standard test for risk assessment in asymptomatic persons. Thus, while a CCTA might be useful for patients with relevant cardiovascular risk factors, no prognostic data are available in this population and thus no clear recommendation can be made. Ongoing studies will help answer this question and may provide needed information to selectively use CCTA for risk assessment. The recently published ASPC clinical practice statement on CCTA provides further information on the role of CCTA in preventive cardiology (230)

## 7 Risk assessment methods in secondary prevention

The term 'secondary prevention' has traditionally been used to describe preventive measures in those with known coronary heart or other ASCVD. The distinction between secondary and primary prevention in those without known disease has become blurred because imaging techniques may reveal atherosclerosis in asymptomatic persons. For the purposes of this paper, the term 'secondary prevention' will be applied to any person with unequivocal evidence of ASCVD whether symptomatic or not. This section focuses on cardiovascular risk assessment in secondary prevention.

Since the middle of the last century when cardiovascular mortality peaked in many developed countries, multiple environmental, lifestyle and treatment factors have contributed to reduced age-standardized coronary heart disease mortality and improved survival after an acute coronary syndrome event (231). However, many persons still suffer from recurrent cardiovascular events due to suboptimal risk factor management, a concept called 'residual risk'. Intensive risk factor and other treatment approaches, some novel and emerging, are focused on addressing this residual risk.

### 7.1 Definitions of risk in secondary prevention

Most guidelines describe those with ASCVD as at very high risk, but current U.S. and European guidelines differ slightly in their definitions. The 2018 U.S. cholesterol guidelines (15) define 'very high risk' as a history of multiple major ASCVD events or 1 major ASCVD event and multiple high-risk conditions. Thus, they are confined to subjects with clinically manifest ASCVD (Table 2).

**Table 2:** **Criteria for Very High Risk Status. Adapted from Grundy et al. (****15****)** Very high-risk status is defined as two or more major ASCVD events or one major ASCVD event and multiple high risk conditions.

**Major ASCVD Events**
- Recent ACS (within the past 12 mo)
- History of MI (other than recent ACS event listed above)
- History of ischemic stroke
- Symptomatic peripheral arterial disease
**High-Risk Conditions**
-Age ≥65 y
- Heterozygous familial hypercholesterolemia
- History of prior coronary artery bypass surgery or percutaneous coronary intervention outside of the major ASCVD event(s)
- Diabetes mellitus
- Hypertension
- CKD (eGFR 15-59 mL/min/1.73 m^2^)
- Current smoking
- Persistently elevated LDL-C (LDL-C ≥100 mg/dL [≥2.6 mmol/L]) despite maximally tolerated statin therapy and ezetimibe
- History of congestive HF

Those with ASCVD who otherwise do not fit one of these criteria are deemed to be “not at very high risk”. Recently published data provide a rationale for this distinction where those defined to be at very high risk have a 3-fold or greater risk of subsequent events as compared those not at very high risk; and the subset of those with a history of two or more major ASCVD events have a 5-fold greater risk (232). Those defined to be at very high risk also have been shown to be among those who benefitted more (greater absolute risk reduction) from PCSK9 therapy (233).

The 2016 ESC Joint Guidelines on the prevention of CVD in clinical practice are quite explicit in defining those with established CVD as being at very high risk and deserving immediate attention to all risk factors (234). For convenience, these risk categories may be simplified:

a) ***Very high risk****:* Documented CVD, diabetes with end organ damage or a major risk factor, severe CKD or a SCORE risk of 10% or more.
b) ***High risk****:* A very high single risk factor, most others with diabetes, moderate CKD or a SCORE risk of 5-9%.
c) ***Moderate risk****:* A SCORE risk of 1-4%; many middle-aged subjects will fall into this category.
d) ***Low risk****:* A SCORE risk of <1%.

The 2019 ESC/European Atherosclerosis Society (EAS) Guidelines for the management of dyslipidemias (235) use the same categories of risk. The LDL-C goal for very high-risk individuals is a reduction of 50% or more and an LDL-C level of < 1.4 mg/dL. In addition, they recommend that a more aggressive LDL-C goal of < 40 mg/dL "may be considered" for those subjects with ASCVD who experience a second vascular event within two years since such patients have declared themselves to be at very high residual risk.

Both the European 2016 prevention (234) and the 2019 lipid guidelines (235) recommend immediate and intensive risk factor management and do not require further risk stratification. As pointed out by Virani and colleagues (236) the European definition of very high risk is wider than the American one, including asymptomatic subjects with objective evidence of ASCVD and other very high-risk individuals. This may expand the number of patients who are candidates for non-statin add-on therapy.

### 7.2 Should further risk assessment be undertaken in those with ASCVD?

While past guidelines have often treated all persons with ASCVD in the same “bucket” with the same therapeutic recommendations, we know risk can vary widely depending on the number and severity of ASCVD events and other high risk conditions (232), justifying the importance of further risk stratification. Moreover, audits such as EUROASPIRE (237) and SURF (238) have reported that risk factor control is inadequate even in subjects with established CHD, and response to therapy may vary widely (239). De Bacquer and colleagues (240) argue that, since risk may vary between patients, there is a need to identify those in whom an even more intensive prevention strategy should be considered. Based on data from a prospective study of 12,484 patients from 27 countries, they derived and externally validated factors associated with adverse outcomes as increasing age, previous hospitalization with stroke, heart failure or percutaneous coronary intervention (PCI), PAD, self-reported diabetes and its glycemic control, higher non-HDL-C, reduced kidney function, symptoms of depression and anxiety and living in a higher risk country and derived a risk calculator based on these findings (240). Whether its use will be associated with improved outcomes remains to be defined. The SMART secondary prevention risk score (241) can also be used to estimate residual risk. It is derived from a low-risk Dutch cohort and may underestimate risk in higher risk countries, an issue that may be addressed by re-calibration.

In 2012, the REACH (REduction of Atherothrombosis for Continued Health) investigators derived a risk model to predict secondary cardiovascular events based on almost 33,419 high-risk subjects from the Americas, Europe, the Middle-East, Asia and Australia (242). An internal validation was performed on 16,270 subjects. Traditional risk factors, burden of disease, lack of treatment, and geographic location were related to an increased risk of subsequent cardiovascular morbidity and cardiovascular mortality. As in EUROASPIRE and SURF, risk factor control was generally poor in all regions. The extent to which this important international study has influenced clinical practice is uncertain.

## 8 Clinical recommendations

1 Assessing a patient's risk for ASCVD is the foundation of preventive cardiology and the initial step for determining the appropriateness and intensity of preventive treatment.
2 In primary prevention, global risk scoring is the initial stage for ASCVD risk assessment, providing a calculation of ASCVD risk from a set of standard office-based risk factors for a specified duration (e.g., 10 years) of time, from which a clinician-patient risk discussion is used to discuss the best ways to reduce CVD risk.
3 The presence, quantity, and/or extent of one or more risk enhancing factors, including premature family history, persistently elevated LDL-C, or CKD, as well as severity of certain inflammatory factors such as hsCRP and laboratory measures such as lp(a), can further inform the treatment decision.
4 In women, it is important to take a comprehensive reproductive history from menarche to menopause, including preeclampsia, premature menopause, and autoimmune disease as “risk-enhancing” factors.
5 Race/ethnicity may have a significant impact on the validity of current risk assessment tools and certain higher risk race/ethnic groups may further inform the use of preventive therapy.
6 Social determinants of health may exert independent effects beyond race/ethnicity and need also to be part of the clinician-patient discussion when discussing the most appropriate ways to optimize ASCVD risk.
7 Among subclinical atherosclerotic disease screening tests, CAC is probably the most useful, providing substantial improvement of risk reclassification over global risk scoring in most primary prevention groups, including diabetes. In addition to the consideration of risk enhancing factors (discussed earlier), CAC testing can be used to further inform treatment decisions for preventive therapy, including statin and aspirin use in particular.
8 The use of ABI for assessment of PAD is also valuable and can improve risk reclassification beyond global risk scoring.
9 Carotid ultrasound imaging, if accompanied by carotid plaque assessment may also be useful for risk assessment, especially as an option when CAC scoring is not available.
10 In patients with pre-existing ASCVD, stratification into those at highest risk (e.g., very high risk ASCVD status) for more aggressive treatment is based on the history of multiple major ASCVD events or one major event and multiple high-risk conditions. Moreover, those with recurrent ASCVD events in the short-term define an extreme risk condition warranting even more aggressive risk factor management.

## 9 Conclusions

Cardiovascular risk assessment is the foundation of preventive cardiology for determining the appropriateness or intensification of preventive therapy. Global risk assessment / scoring is the first step in cardiovascular risk assessment, after which the presence of other risk enhancing factors, inflammatory and other biomarkers, sex, ethnic, and social determinants of health, and screening for subclinical atherosclerosis can further refine risk estimation. Moreover, nutritional factors and physical activity and fitness levels (addressed in other ASPC clinical practice statements) should be considered. In secondary prevention those at very high risk can be identified on the basis of the presence of multiple major ASCVD events and/or high-risk conditions where more intensive treatment may be warranted. Ultimately, ASCVD risk assessment remains an inexact science and while it can be useful for assessing risk in populations of subjects, application to the individual patient is still limited; however, novel emerging methods to incorporate sociodemographic, genetic, clinical, and lifestyle measures will hopefully improve precision for risk prediction for the individual patient.

## Authorship Contributions

All authors contributed to the writing of the manuscript and provided critical revision and review and approval of submission.

## Funding

No funding was received for the preparation of this manuscript.

## Author Disclosures

Dr. Michos reports advisory board participation for Astra Zeneca, Amarin, Bayer, Boehringer Ingelheim, Esperion, Novo Nordisk, and Novartis, Dr Shapiro reports advisory board participation for Amgen, Novartis, and Novo Nordisk, and consulting for Novartis and Regeneron, and Dr. Wong reports research funding through his institution from Novartis and Gilead, consultant for Novartis, and advisory board for Amgen. Dr. Amit Khera served as guest editor for this manuscript and Drs. Wong and Michos had no role in the review or decision process.

## Declaration of Competing Interest

None

## References

[1] Kannel WB, Dawber TR, Kagan A, Revotskie N (1961). Strokes J 3rd. Factors of risk in the development of coronary heart disease–six year follow-up experience. The Framingham Study. Ann Intern Med.

[2] Truett J, Cornfield J, Kannel WB. (1967). A multivariate analysis of the risk of coronary heart disease in Framingham. J Chronic Dis.

[3] Kannel WB, McGee D, Gordon T. (1976). A general cardiovascular risk profile: the Framingham Study. Am J Cardiol.

[4] Califf RM, Armstrong PW, Carver JR, D'Agostino RB, Strauss WE. (1996). 27th Bethesda Conference: matching the intensity of risk factor management with the hazard for coronary disease events. Task Force 5. Stratification of patients into high, medium and low risk subgroups for purposes of risk factor management. J Am Coll Cardiol.

[5] Pignone M, Phillips CJ, Elasy TA, Fernandez A. (2003). Physicians' ability to predict the risk of coronary heart disease. BMC Health Serv Res.

[6] Sheridan SL, Crespo E. (2008). Does the routine use of global coronary heart disease risk scores translate into clinical benefits or harms? A systematic review of the literature. BMC Health Serv Res.

[7] Anderson KM, Wilson PW, Odell PM, Kannel WB. (1991). An updated coronary risk profile. A statement for health professionals. Circulation.

[8] Detection Expert Panel on, Evaluation and (2001). Treatment of High Blood Cholesterol in Adults. Executive Summary of The Third Report of The National Cholesterol Education Program (NCEP) Expert Panel on Detection, Evaluation, And Treatment of High Blood Cholesterol In Adults (Adult Treatment Panel III). JAMA.

[9] Sr D'Agostino RB, Pencina MJ, Massaro JM, Coady S. (2013). Cardiovascular Disease Risk Assessment: Insights from Framingham. Glob Heart.

[10] Sr D'Agostino RB, Vasan RS, Pencina MJ (2008). General cardiovascular risk profile for use in primary care: the Framingham Heart Study. Circulation.

[11] Conroy RM, Pyörälä K, Fitzgerald AP (2003). Estimation of ten-year risk of fatal cardiovascular disease in Europe: the SCORE project. Eur Heart J.

[12] (2021). SCORE2 working group and ESC Cardiovascular risk collaboration. SCORE2 risk prediction algorithms: new models to estimate 10-year risk of cardiovascular disease in Europe. Eur Heart J.

[13] Mach F, Baigent C, Catapano AL (2020). ESC Scientific Document Group, 2019 ESC/EAS Guidelines for the management of dyslipidaemias: *lipid modification to reduce cardiovascular risk*: The Task Force for the management of dyslipidaemias of the European Society of Cardiology (ESC) and European Atherosclerosis Society (EAS). Eur Heart J.

[14] WHO CVD Risk Chart Working Group (2019 Oct). World Health Organization cardiovascular disease risk charts: revised models to estimate risk in 21 global regions. Lancet Glob Health.

[15] Grundy SM, Stone NJ, Bailey AL (2019). 2018 AHA/ACC/AACVPR/AAPA/ABC/ACPM/ADA/AGS/APhA/ASPC/NLA/PCNA Guideline on the Management of Blood Cholesterol: A Report of the American College of Cardiology/American Heart Association Task Force on Clinical Practice Guidelines. J Am Coll Cardiol.

[16] Arnett DK, Blumenthal RS, Albert MA (2019). 2019 ACC/AHA Guideline on the Primary Prevention of Cardiovascular Disease: A Report of the American College of Cardiology/American Heart Association Task Force on Clinical Practice Guidelines. Am Coll Cardiol.

[17] Lloyd-Jones DM, Braun LT, Ndumele CE, Jr Smith SC, Sperling LS, Virani SS, Blumenthal RS (2019). Use of Risk Assessment Tools to Guide Decision-Making in the Primary Prevention of Atherosclerotic Cardiovascular Disease: A Special Report From the American Heart Association and American College of Cardiology. J Am Coll Cardiol.

[18] Whelton PK, Carey RM, Aronow WS (2018). 2017 ACC/AHA/AAPA/ABC/ACPM/AGS/APhA/ASH/ASPC/NMA/PCNA Guideline for the Prevention, Detection, Evaluation, and Management of High Blood Pressure in Adults: A Report of the American College of Cardiology/American Heart Association Task Force on Clinical Practice Guidelines. J Am Coll Cardiol.

[19] Bulugahapitiya U, Siyambalapitiya S, Sithole J, Idris I. (2009 Feb). Is diabetes a coronary risk equivalent? Systematic review and meta-analysis. Diabet Med.

[20] Wong ND, Glovaci D, Wong K, Malik S, Franklin SS, Wygant G, Iloeje U. (2012). Global cardiovascular disease risk assessment in United States adults with diabetes. Diab Vasc Dis Res.

[21] United Kingdom Prospective Diabetes Study risk engine http://www.dtu.ox.ac.uk/riskengine.

[22] Yeboah J, Erbel R, Delaney JC (2014). Development of a new diabetes risk prediction tool for incident coronary heart disease events: the Multi-Ethnic Study of Atherosclerosis and the Heinz Nixdorf Recall Study. Atherosclerosis..

[23] Ridker PM, Buring JE, Rifai N, Cook NR. (2007). Development and validation of improved algorithms for the assessment of global cardiovascular risk in women: the Reynolds Risk Score. JAMA.

[24] Khera AV, Emdin CA, Drake I (2016). Genetic Risk, Adherence to a Healthy Lifestyle, and Coronary Disease. N Engl J Med.

[25] Aragam KG, Dobbyn A, Judy R (2020). Limitations of Contemporary Guidelines for Managing Patients at High Genetic Risk of Coronary Artery Disease. J Am Coll Cardiol.

[26] Javed Z, Valero-Elizondo J, Dudum R, Khan SU, Dubey P, Hyder AA, Xu J, Bilal U, Kash BA, Cainzos-Achirica M, Nasir K. (2021). Development and validation of a polysocial risk score for atherosclerotic cardiovascular disease. Am J Prev Cardiol.

[27] Martin SS, Sperling LS, Blaha MJ, Wilson PWF, Gluckman TJ, Blumenthal RS, Stone NJ. (2015 Apr 7). Clinician-patient risk discussion for atherosclerotic cardiovascular disease prevention: importance to implementation of the 2013 ACC/AHA Guidelines. J Am Coll Cardiol.

[28] Lloyd-Jones DM, Nam BH, D'Agostino RB (2004). Parental Cardiovascular Disease as a Risk Factor for Cardiovascular Disease in Middle-aged Adults: A Prospective Study of Parents and Offspring. J Am Med Assoc.

[29] Moonesinghe R, Yang Q, Zhang Z, Khoury MJ. (2019). Prevalence and Cardiovascular Health Impact of Family History of Premature Heart Disease in the United States: Analysis of the National Health and Nutrition Examination Survey, 2007–2014. J Am Heart Assoc.

[30] Ference BA, Ginsberg HN, Graham I (2017). Low-density lipoproteins cause atherosclerotic cardiovascular disease. 1. Evidence from genetic, epidemiologic, and clinical studies. A consensus statement from the European Atherosclerosis Society Consensus Panel. Eur Heart J.

[31] Giugliano RP, Pedersen TR, Park JG (2017). Clinical efficacy and safety of achieving very low LDL-cholesterol concentrations with the PCSK9 inhibitor evolocumab: a prespecified secondary analysis of the FOURIER trial. Lancet.

[32] Alberti KG, Eckel RH, Grundy SM (2009). Harmonizing the metabolic syndrome: a joint interim statement of the International Diabetes Federation Task Force on Epidemiology and Prevention; National Heart, Lung, and Blood Institute; American Heart Association; World Heart Federation; International Atherosclerosis Society; and International Association for the Study of Obesity. Circulation.

[33] Shin D, Kongpakpaisarn K, Bohra C. (2018). Trends in the prevalence of metabolic syndrome and its components in the United States 2007-2014. Int J Cardiol.

[34] Lahey R, Khan SS. (2018). Trends in obesity and risk of cardiovascular disease. Current Epidemiology Reports.

[35] Hansildaar R, Vedder D, Baniaamam M, Tausche AK, Gerritsen M, Nurmohamed MT. (2021). Cardiovascular risk in inflammatory arthritis: rheumatoid arthritis and gout. Lancet Rheumatol.

[36] Avina-Zubieta JA, Choi HK, Sadatsafavi M, Etminan M, Esdaile JM, Lacaille D. (2008). Risk of cardiovascular mortality in patients with rheumatoid arthritis: a meta-analysis of observational studies. Arthritis Rheum.

[37] Asanuma Y, Oeser A, Shintani AK (2003). Premature coronary-artery atherosclerosis in systemic lupus erythematosus. N Engl J Med.

[38] Nossent J, Cikes N, Kiss E (2007). Current causes of death in systemic lupus erythematosus in Europe, 2000–2004: relation to disease activity and damage accrual. Lupus.

[39] Armstrong EJ, Harskamp CT, Armstrong AW. (2013). Psoriasis and major adverse cardiovascular events: a systematic review and meta-analysis of observational studies. J Am Heart Assoc.

[40] Kearns A, Gordon J, Burdo TH, Qin X (2017). HIV-1-Associated Atherosclerosis: Unraveling the Missing Link. J Am Coll Cardiol.

[41] Saran R, Robinson B, Abbott KC (2019). US Renal Data System 2018 Annual Data Report: Epidemiology of Kidney Disease in the United States. AJKD.

[42] Babitt JL, Lin HY. (2012). Mechanisms of anemia in CKD. J Am Soc Nephrol.

[43] Toussaint ND, Pedagogos E, Lioufas NM (2020). A Randomized Trial on the Effect of Phosphate Reduction on Vascular End Points in CKD (IMPROVE-CKD). JASN.

[44] Boden WE, Probstfield JL, Anderson T (2011). Niacin in patients with low HDL cholesterol levels receiving intensive statin therapy. N Engl J Med.

[45] Bhatt DL, Steg G, Miller M (2019). Cardiovascular Risk Reduction with Icosapent Ethyl for. Hypertriglyceridemia N Engl J Med.

[46] Albers JJ, Kennedy H, Marcovina SM. (1996). Evidence that Lp[a] contains one molecule of apo[a] and one molecule of apoB: evaluation of amino acid analysis data. J Lipid Res.

[47] Danesh J, Collins R, Peto R. (2000). Lipoprotein(a) and coronary heart disease. Meta-analysis of prospective studies. Circulation.

[48] Kamstrup PR, Tybjaerg-Hansen A, Steffensen R, Nordestgaard BG. (2009). Genetically elevated lipoprotein(a) and increased risk of myocardial infarction. JAMA.

[49] Eaton DL, Fless GM, Kohr WJ (1987). Partial amino acid sequence of apolipoprotein(a) shows that it is homologous to plasminogen. Proc Natl Acad Sci U S A.

[50] Willeit P, Kiechl F., Kronenberg F (2014). Discrimination and net reclassification of cardiovascular risk with lipoprotein(a): prospective 15-year outcomes in the Bruneck Study. J Am Coll Cardiol.

[51] Sniderman AD, Williams K, Contois JH (2011). A meta-analysis of low-density lipoprotein cholesterol, non-high-density lipoprotein cholesterol, and apolipoprotein B as markers of cardiovascular risk. Circ Cardiovasc Qual Outcomes.

[52] Sniderman AD, Tremblay A, De Graaf J (2012). Phenotypes of hypertriglyceridemia caused by excess very-low-density lipoprotein. J Clin Lipidol.

[53] Wang TJ, Gona P, Larson MG (2006). Multiple biomarkers for the prediction of first major cardiovascular events and death. N Engl J Med.

[54] Di Angelantonio E, Chowdhury R, Sarwar N (2009). B-type natriuretic peptides and cardiovascular risk: systematic review and meta-analysis of 40 prospective studies. Circulation.

[55] Greenland P, Alpert JS, Beller GA (2010 Dec 21). American College of Cardiology Foundation/American Heart Association Task Force on Practice Guidelines. 2010 ACCF/AHA guideline for assessment of cardiovascular risk in asymptomatic adults: a report of the American College of Cardiology Foundation/American Heart Association Task Force on Practice Guidelines. Circulation.

[56] Buchan TA, Ching C, Foroutan F (2021). Prognostic value of natriuretic peptides in heart failure: systematic review and meta-analysis. Heart Fail Rev.

[57] Yancy CW, Jessup M, Bozkurt B (2017). 2017 ACC/AHA/HFSA Focused Update of the 2013 ACCF/AHA Guideline for the Management of Heart Failure: A Report of the American College of Cardiology/American Heart Association Task Force on Clinical Practice Guidelines and the Heart Failure Society of America. J Am Coll Cardiol.

[58] Otto CM, Nishimura RA, Bonow RO (2021). 2020 ACC/AHA Guideline for the Management of Patients With Valvular Heart Disease: A Report of the American College of Cardiology/American Heart Association Joint Committee on Clinical Practice Guidelines. J Am Coll Cardiol.

[59] Roffi M, Patrono C, Collet JP (2016). ESC Scientific Document Group. 2015 ESC Guidelines for the management of acute coronary syndromes in patients presenting without persistent ST-segment elevation: Task Force for the Management of Acute Coronary Syndromes in Patients Presenting without Persistent ST-Segment Elevation of the European Society of Cardiology (ESC). Eur Heart J.

[60] Passino C, Aimo A, Masotti S (2019). Cardiac troponins as biomarkers for cardiac disease. Biomark Med.

[61] Lam CSP, Castillo R, Ho DT (2019). High-sensitivity troponin I for cardiovascular risk stratification in the general asymptomatic population: Perspectives from Asia-Pacific. Int J Cardiol.

[62] Furman D, Campisi J, Verdin E (2019). Chronic inflammation in the etiology of disease across the life span. Nat Med.

[63] Libby P. (2012). Inflammation in atherosclerosis. Arterioscler Thromb Vasc Biol.

[64] Libby P, Ridker PM, Hansson GK. (2009). Inflammation in atherosclerosis: from pathophysiology to practice. J Am Coll Cardiol.

[65] Ross R. (1999). Atherosclerosis–an inflammatory disease. N Engl J Med.

[66] Chen L, Deng H, Cui H (2018). Inflammatory responses and inflammation-associated diseases in organs. Oncotarget.

[67] Libby P. (2005). Act local, act global: inflammation and the multiplicity of "vulnerable" coronary plaques. J Am Coll Cardiol.

[68] Campbell KA, Lipinski MJ, Doran AC, Skaflen MD, Fuster V, McNamara CA. (2012). Lymphocytes and the adventitial immune response in atherosclerosis. Circ Res.

[69] Bujo H, Saito Y. (2006). Modulation of smooth muscle cell migration by members of the low-density lipoprotein receptor family. Arterioscler Thromb Vasc Biol.

[70] Sproston NR, Ashworth JJ. (2018). Role of C-Reactive Protein at Sites of Inflammation and Infection. Front Immunol.

[71] Ridker PM (2003). C-Reactive Protein. Circulation.

[72] Wilson PW, Pencina M, Jacques P, Selhub J, D'Agostino R, O'Donnell CJ (2008). C-reactive protein and reclassification of cardiovascular risk in the Framingham Heart Study. Circ Cardiovasc Qual Outcomes.

[73] Ridker PM, Paynter NP, Rifai N, Gaziano JM (2008). Cook NR. C-reactive protein and parental history improve global cardiovascular risk prediction: the Reynolds Risk Score for men. Circulation.

[74] Ridker PM, Rifai N, Rose L, Buring JE, Cook NR. (2002). Comparison of C-reactive protein and low-density lipoprotein cholesterol levels in the prediction of first cardiovascular events. N Engl J Med.

[75] Ridker PM, Cannon CP, Morrow D (2005). C-reactive protein levels and outcomes after statin therapy. N Engl J Med.

[76] Morrow DA, de Lemos JA, Sabatine MS (2006). Clinical relevance of C-reactive protein during follow-up of patients with acute coronary syndromes in the Aggrastat-to-Zocor Trial. Circulation.

[77] Bohula EA, Giugliano RP, Cannon CP (2015). Achievement of dual low-density lipoprotein cholesterol and high-sensitivity C-reactive protein targets more frequent with the addition of ezetimibe to simvastatin and associated with better outcomes in IMPROVE-IT. Circulation.

[78] Ridker PM, Rifai N, Clearfield M (2001). Measurement of C-reactive protein for the targeting of statin therapy in the primary prevention of acute coronary events. N Engl J Med.

[79] Gonçalves I, Edsfeldt A, Ko NY (2012). Evidence supporting a key role of Lp-PLA2-generated lysophosphatidylcholine in human atherosclerotic plaque inflammation. Arterioscler Thromb Vasc Biol.

[80] Kolodgie FD, Burke AP, Skorija KS (2006). Lipoprotein-associated phospholipase A2 protein expression in the natural progression of human coronary atherosclerosis. Arterioscler Thromb Vasc Biol.

[81] Silva IT, Mello AP, Damasceno NR. (2011). Antioxidant and inflammatory aspects of lipoprotein-associated phospholipase A₂ (Lp-PLA₂): a review. Lipids Health Dis.

[82] Garza CA, Montori VM, McConnell JP, Somers VK, Kullo IJ, Lopez-Jimenez F. (2007). Association between lipoprotein-associated phospholipase A2 and cardiovascular disease: a systematic review. Mayo Clin Proc.

[83] Ballantyne CM, Hoogeveen RC, Bang H (2004). Lipoprotein-associated phospholipase A2, high-sensitivity C-reactive protein, and risk for incident coronary heart disease in middle-aged men and women in the Atherosclerosis Risk in Communities (ARIC) study. Circulation.

[84] White HD, Held C, Stewart R (2014). Darapladib for preventing ischemic events in stable coronary heart disease. N Engl J Med.

[85] Aratani Y. (2018). Myeloperoxidase: Its role for host defense, inflammation, and neutrophil function. Arch Biochem Biophys.

[86] Shao B, Oda MN, Oram JF, Heinecke JW. (2010). Myeloperoxidase: an oxidative pathway for generating dysfunctional high-density lipoprotein. Chem Res Toxicol.

[87] Brennan ML, Penn MS, Van Lente F (2003). Prognostic value of myeloperoxidase in patients with chest pain. N Engl J Med.

[88] Baldus S, Heeschen C, Meinertz T (2003). Myeloperoxidase serum levels predict risk in patients with acute coronary syndromes. Circulation.

[89] Yang D, Han Z, Oppenheim JJ. (2017). Alarmins and immunity. Immunol Rev.

[90] Sakuma M, Tanaka A, Kotooka N (2017). Myeloid-related protein-8/14 in acute coronary syndrome. Int J Cardiol.

[91] Croce K, Gao H, Wang Y (2009). Myeloid-Related Protein-8/14 Is Critical for the Biological Response to Vascular Injury. Circulation.

[92] Maiseyeu A, Badgeley MA, Kampfrath T (2012). In vivo targeting of inflammation-associated myeloid-related protein 8/14 via gadolinium immunonanoparticles. Arterioscler Thromb Vasc Biol.

[93] Morrow DA, Wang Y, Croce K (2008). Myeloid-related protein 8/14 and the risk of cardiovascular death or myocardial infarction after an acute coronary syndrome in the Pravastatin or Atorvastatin Evaluation and Infection Therapy: Thrombolysis in Myocardial Infarction (PROVE IT-TIMI 22) trial. Am Heart J.

[94] Peng WH, Jian WX, Li HL (2011). Increased serum myeloid-related protein 8/14 level is associated with atherosclerosis in type 2 diabetic patients. Cardiovascular Diabetology.

[95] Visse R, Nagase H. (2003). Matrix Metalloproteinases and Tissue Inhibitors of Metalloproteinases. Circ Res.

[96] Volkov AM, Murashov IS, Polonskaya YV (2018). [Changes of Content of Matrix Metalloproteinases and Their Tissue Expression in Various Types of Atherosclerotic Plaques]. Kardiologiia.

[97] Ezhov M, Safarova M, Afanasieva O, Mitroshkin M, Matchin Y, Pokrovsky S. (2019). Matrix Metalloproteinase 9 as a Predictor of Coronary Atherosclerotic Plaque Instability in Stable Coronary Heart Disease Patients with Elevated Lipoprotein(a) Levels. Biomolecules.

[98] Lenglet S, Mach F, Montecucco F. (2013). Role of matrix metalloproteinase-8 in atherosclerosis. Mediators Inflamm.

[99] Leopold JA, Loscalzo J. (2005). Oxidative enzymopathies and vascular disease. Arterioscler Thromb Vasc Biol.

[100] Barreto J, Karathanasis SK, Remaley A, Sposito AC. (2021). Role of LOX-1 (Lectin-Like Oxidized Low-Density Lipoprotein Receptor 1) as a Cardiovascular Risk Predictor: Mechanistic Insight and Potential Clinical Use. Arterioscler Thromb Vasc Biol.

[101] Lubrano V, Del Turco S, Nicolini G, Di Cecco P, Basta G. (2008). Circulating Levels of Lectin-Like Oxidized Low-Density Lipoprotein Receptor-1 are Associated with Inflammatory Markers. Lipids.

[102] Zhao ZW, Zhu XL, Luo YK, Lin CG, Chen LL. (2011). Circulating soluble lectin-like oxidized low-density lipoprotein receptor-1 levels are associated with angiographic coronary lesion complexity in patients with coronary artery disease. Clin Cardiol.

[103] Wischhusen J, Melero I, Fridman WH. (2020). Growth/Differentiation Factor-15 (GDF-15): From Biomarker to Novel Targetable Immune Checkpoint. Front Immunol.

[104] Wang J, Wei L, Yang X, Zhong J. (2019). Roles of Growth Differentiation Factor 15 in Atherosclerosis and Coronary Artery Disease. J Am Heart Assoc.

[105] Wollert KC, Kempf T, Wallentin L. (2017). Growth Differentiation Factor 15 as a Biomarker in Cardiovascular Disease. Clin Chem.

[106] Ridker PM, Everett BM, Thuren T (2017). CANTOS Trial Group. Antiinflammatory Therapy with Canakinumab for Atherosclerotic Disease. N Engl J Med.

[107] Gabay C, Lamacchia C, Palmer G. (2010). IL-1 pathways in inflammation and human diseases. Nature Reviews Rheumatology.

[108] Guo H, Callaway JB, Ting JP. (2015). Inflammasomes: mechanism of action, role in disease, and therapeutics. Nat Med.

[109] Tardif JC, Kouz S, Waters DD (2019). Efficacy and Safety of Low-Dose Colchicine after Myocardial Infarction. N Engl J Med.

[110] Virani SS, Alonso A, Aparicio HJ (2021). Heart Disease and Stroke Statistics-2021 Update: A Report From the. American Heart Association. Circulation..

[111] Mosca L, Grundy SM, Judelson D (1999). Guide to Preventive Cardiology for Women.AHA/ACC Scientific Statement Consensus panel statement. Circulation.

[112] Curtin SC. (2019). Trends in Cancer and Heart Disease Death Rates Among Adults Aged 45-64: United States, 1999-2017. Natl Vital Stat Rep.

[113] Khan SU, Yedlapati SH, Lone AN (2021 Feb 8). A comparative analysis of premature heart disease- and cancer-related mortality in women in the USA, 1999-2018. Eur Heart J Qual Care Clin Outcomes.

[114] Cushman M, Shay CM, Howard VJ (2021). Ten-Year Differences in Women's Awareness Related to Coronary Heart Disease: Results of the 2019 American Heart Association National Survey: A Special Report From the American Heart Association. Circulation..

[115] Mannoh I, Hussien M, Commodore-Mensah Y (2021). Impact of social determinants of health on cardiovascular disease prevention. Curr Opin Cardiol.

[116] DeFilippis AP, Young R, McEvoy JW (2017). Risk score overestimation: the impact of individual cardiovascular risk factors and preventive therapies on the performance of the American Heart Association-American College of Cardiology-Atherosclerotic Cardiovascular Disease risk score in a modern multi-ethnic cohort. Eur Heart J.

[117] Brindle PM, McConnachie A, Upton MN (2005). The accuracy of the Framingham risk-score in different socioeconomic groups: a prospective study. Br J Gen Pract.

[118] Amin NP, Martin SS, Blaha MJ (2014). Headed in the right direction but at risk for miscalculation: a critical appraisal of the 2013 ACC/AHA risk assessment guidelines. J Am Coll Cardiol.

[119] Michos ED, Nasir K, Braunstein JB (2006). Framingham risk equation underestimates subclinical atherosclerosis risk in asymptomatic women. Atherosclerosis.

[120] Michos ED, Vasamreddy CR, Becker DM (2005). Women with a low Framingham risk score and a family history of premature coronary heart disease have a high prevalence of subclinical coronary atherosclerosis. Am Heart J.

[121] Damen JA, Pajouheshnia R, Heus P (2019). Performance of the Framingham risk models and pooled cohort equations for predicting 10-year risk of cardiovascular disease: a systematic review and meta-analysis. BMC Med.

[122] Grundtvig M, Hagen TP, German M (2009). Sex-based differences in premature first myocardial infarction caused by smoking: twice as many years lost by women as by men. Eur J Cardiovasc Prev Rehabil.

[123] Peters SA, Huxley RR, Woodward M. (2014). Diabetes as risk factor for incident coronary heart disease in women compared with men: a systematic review and meta-analysis of 64 cohorts including 858,507 individuals and 28,203 coronary events. Diabetologia.

[124] Fairweather D, Rose NR. (2004). Women and autoimmune diseases. Emerg Infect Dis.

[125] del Rincon ID, Williams K, Stern MP (2001). High incidence of cardiovascular events in a rheumatoid arthritis cohort not explained by traditional cardiac risk factors. Arthritis Rheum.

[126] Avina-Zubieta JA, Choi HK, Sadatsafavi M (2008). Risk of cardiovascular mortality in patients with rheumatoid arthritis: a meta-analysis of observational studies. Arthritis Rheum.

[127] Hansildaar R, Vedder D, Baniaamam M (2021). Cardiovascular risk in inflammatory arthritis: rheumatoid arthritis and gout. Lancet Rheumatol.

[128] Avina-Zubieta JA, To F, Vostretsova K (2017). Risk of Myocardial Infarction and Stroke in Newly Diagnosed Systemic Lupus Erythematosus: A General Population-Based Study. Arthritis Care Res (Hoboken).

[129] Giannelou M, Mavragani CP. (2017). Cardiovascular disease in systemic lupus erythematosus: A comprehensive update. J Autoimmun.

[130] Asanuma Y, Oeser A, Shintani AK (2003). Premature coronary-artery atherosclerosis in systemic lupus erythematosus. N Engl J Med.

[131] Chung CP, Oeser A, Raggi P (2005). Increased coronary-artery atherosclerosis in rheumatoid arthritis: relationship to disease duration and cardiovascular risk factors. Arthritis Rheum.

[132] Agarwala A, Michos ED, Samad Z (2020). The Use of Sex-Specific Factors in the Assessment of Women's Cardiovascular Risk. Circulation.

[133] Elder P, Sharma G, Gulati M (2020). Identification of female-specific risk enhancers throughout the lifespan of women to improve cardiovascular disease prevention. Am J Prev Cardiol.

[134] Peters SA, Woodward M. (2018). Women's reproductive factors and incident cardiovascular disease in the UK Biobank. Heart.

[135] Okoth K, Chandan JS, Marshall T (2020). Association between the reproductive health of young women and cardiovascular disease in later life: umbrella review. BMJ.

[136] Lakshman R, Forouhi NG, Sharp SJ (2009). Early age at menarche associated with cardiovascular disease and mortality. J Clin Endocrinol Metab.

[137] Osibogun O, Ogunmoroti O, Michos ED. (2020). Polycystic ovary syndrome and cardiometabolic risk: Opportunities for cardiovascular disease prevention. Trends Cardiovasc Med.

[138] Goueslard K, Cottenet J, Mariet AS (2016). Early cardiovascular events in women with a history of gestational diabetes mellitus. Cardiovasc Diabetol.

[139] de Groot PC, Dekkers OM, Romijn JA (2011). PCOS, coronary heart disease, stroke and the influence of obesity: a systematic review and meta-analysis. Hum Reprod Update.

[140] Pomp ER, Rosendaal FR, Doggen CJ. (2008). Smoking increases the risk of venous thrombosis and acts synergistically with oral contraceptive use. Am J Hematol.

[141] Lidegaard O. (1999). Smoking and use of oral contraceptives: impact on thrombotic diseases. Am J Obstet Gynecol.

[142] Cairncross ZF, Ahmed SB, Dumanski SM (2021). Infertility and the Risk of Cardiovascular Disease: Findings From the Study of Women's Health Across the Nation (SWAN). CJC Open.

[143] Westerlund E, Brandt L, Hovatta O (2014). Incidence of hypertension, stroke, coronary heart disease, and diabetes in women who have delivered after in vitro fertilization: a population-based cohort study from Sweden. Fertil Steril.

[144] Dayan N, Filion KB, Okano M (2017). Cardiovascular Risk Following Fertility Therapy: Systematic Review and Meta-Analysis. J Am Coll Cardiol.

[145] Udell JA, Lu H, Redelmeier DA. (2017). Failure of fertility therapy and subsequent adverse cardiovascular events. CMAJ.

[146] Peters SA, van der Schouw YT, Wood AM (2016). Parity, breastfeeding and risk of coronary heart disease: A pan-European case-cohort study. Eur J Prev Cardiol.

[147] Oliver-Williams C, Vladutiu CJ, Loehr LR (2019). The Association Between Parity and Subsequent Cardiovascular Disease in Women: The Atherosclerosis Risk in Communities Study. J Women's Health (2002).

[148] Ness RB, Harris T, Cobb J (1993). Number of pregnancies and the subsequent risk of cardiovascular disease. N Engl J Med.

[149] Mazaki-Tovi S, Vaisbuch EDI, Romero R. (2013). Adipokines and pathophysiology of pregnancy complications - the role of leptin and adiponectin. Fetal Maternal Med Rev.

[150] Rodriguez CP, Ogunmoroti O, Quispe R (2020). Abstract 13525: The Association Between Multiparity and Adipokine Levels: The Multi-Ethnic Study of Atherosclerosis (MESA). Circulation.

[151] Bond RM, Gaither K, Nasser SA (2021). Working Agenda for Black Mothers: A Position Paper From the Association of Black Cardiologists on Solutions to Improving Black Maternal Health. Circ Cardiovasc Qual Outcomes.

[152] Minhas AS, Ogunwole SM, Vaught AJ (2021). Racial Disparities in Cardiovascular Complications With Pregnancy-Induced Hypertension in the United States. Hypertension (Dallas, Tex: 1979).

[153] Hauspurg A, Ying W, Hubel CA (2018). Adverse pregnancy outcomes and future maternal cardiovascular disease. Clin Cardiol.

[154] Ying W, Catov JM, Ouyang P. (2018). Hypertensive Disorders of Pregnancy and Future Maternal Cardiovascular Risk. J Am Heart Assoc.

[155] Wu P, Haththotuwa R, Kwok CS (2017). Preeclampsia and Future Cardiovascular Health: A Systematic Review and Meta-Analysis. Circ Cardiovasc Qual Outcomes.

[156] Heida KY, Velthuis BK, Oudijk MA (2016). Cardiovascular disease risk in women with a history of spontaneous preterm delivery: A systematic review and meta-analysis. Eur J Prev Cardiol.

[157] Damm P, Houshmand-Oeregaard A, Kelstrup L (2016). Gestational diabetes mellitus and long-term consequences for mother and offspring: a view from Denmark. Diabetologia.

[158] Fadl H, Magnuson A, Ostlund I (2014). Gestational diabetes mellitus and later cardiovascular disease: a Swedish population based case-control study. BJOG.

[159] Kramer CK, Campbell S, Retnakaran R. (2019). Gestational diabetes and the risk of cardiovascular disease in women: a systematic review and meta-analysis. Diabetologia.

[160] Honigberg MC, Zekavat SM, Aragam K (2019). Association of Premature Natural and Surgical Menopause With Incident Cardiovascular Disease. JAMA.

[161] Muka T, Oliver-Williams C, Kunutsor S (2016). Association of Age at Onset of Menopause and Time Since Onset of Menopause With Cardiovascular Outcomes, Intermediate Vascular Traits, and All-Cause Mortality: A Systematic Review and Meta-analysis. JAMA Cardiol.

[162] Roeters van Lennep JE, Heida KY, Bots ML (2016). Cardiovascular disease risk in women with premature ovarian insufficiency: A systematic review and meta-analysis. Eur J Prev Cardiol.

[163] Tao XY, Zuo AZ, Wang JQ (2016). Effect of primary ovarian insufficiency and early natural menopause on mortality: a meta-analysis. Climacteric.

[164] Subramanya V, Zhao D, Ouyang P (2019). Association of endogenous sex hormone levels with coronary artery calcium progression among post-menopausal women in the Multi-Ethnic Study of Atherosclerosis (MESA). J Cardiovasc Comput Tomogr.

[165] Zhao D, Guallar E, Ballantyne CM (2020). Sex Hormones and Incident Heart Failure in Men and Postmenopausal Women: The Atherosclerosis Risk in Communities Study. J Clin Endocrinol Metab.

[166] Thurston RC, Aslanidou Vlachos HE, Derby CA (2021). Menopausal Vasomotor Symptoms and Risk of Incident Cardiovascular Disease Events in SWAN. J Am Heart Assoc.

[167] Marma AK, Berry JD, Ning H (2010). Distribution of 10-year and lifetime predicted risks for cardiovascular disease in US adults: findings from the National Health and Nutrition Examination Survey 2003 to 2006. Circ Cardiovasc Qual Outcomes.

[168] Wilkins JT, Ning H, Berry J (2012). Lifetime risk and years lived free of total cardiovascular disease. JAMA.

[169] Leppert MH, Ho PM, Burke J (2020). Young Women Had More Strokes Than Young Men in a Large, United States Claims Sample. Stroke.

[170] Elgendy IY, Nadeau SE, Bairey Merz CN (2019). Migraine Headache: An Under-Appreciated Risk Factor for Cardiovascular Disease in Women. J Am Heart Assoc.

[171] Markovitz AR, Stuart JJ, Horn J (2019). Does pregnancy complication history improve cardiovascular disease risk prediction? findings from the HUNT study in Norway. Eur Heart J.

[172] Michos ED, Blaha MJ, Blumenthal RS. (2017). Use of the coronary artery calcium score in discussion of initiation of statin therapy in primary prevention. Mayo Clin Proc.

[173] Budoff MJ, Young R, Burke G (2018). Ten-year association of coronary artery calcium with atherosclerotic cardiovascular disease (ASCVD) events: the multi-ethnic study of atherosclerosis (MESA). Eur Heart J.

[174] Kavousi M, Desai CS, Ayers C (2016). Prevalence and Prognostic Implications of Coronary Artery Calcification in Low-Risk Women: A Meta-analysis. JAMA.

[175] Lakoski SG, Greenland P, Wong ND (2007). Coronary artery calcium scores and risk for cardiovascular events in women classified as "low risk" based on Framingham risk score: the multi-ethnic study of atherosclerosis (MESA). Arch Intern Med.

[176] Shaw LJ, Min JK, Nasir K (2018). Sex differences in calcified plaque and long-term cardiovascular mortality: observations from the CAC Consortium. Eur Heart J.

[177] Wong ND, Cordola Hsu AR, Rozanski A (2020). Sex Differences in Coronary Artery Calcium and Mortality From Coronary Heart Disease, Cardiovascular Disease, and All Causes in Adults With Diabetes: The Coronary Calcium Consortium. Diabetes Care.

[178] (March 31, 2021).

[179] Flanagin A, Frey T, Christiansen SL, Bauchner H. (2021). The Reporting of Race and Ethnicity in Medical and Science Journals: Comments Invited. JAMA.

[180] Kochanek KD (2019). National Vital Statistic Report.

[181] Virani SS, Alonso A, Aparicio HJ (2021 Feb 23). American heart association council on epidemiology and prevention statistics committee and stroke statistics subcommittee. heart disease and stroke statistics-2021 update: a report From the. Am Heart Assoc Circul.

[182] Anderson KM, Odell PM, Wilson PWF, Kannel WB. (1991). Cardiovascular disease risk profiles. Am Heart J.

[183] Wilson PWF, Castelli WP, Kannel WB. (1987). Coronary risk prediction in adults (The Framingham Heart Study). Am J Cardiol.

[184] D'Agostino RB, Grundy S, Sullivan LM, Wilson P (2001). Validation of the Framingham coronary heart disease prediction scores: Results of a multiple ethnic groups investigation. JAMA.

[185] Andersson C, Nayor M, Tsao CW, Levy D, Vasan RS. (2021). Framingham Heart Study. J Am Coll Cardiol.

[186] Colantonio LD, Bittner V, Reynolds K (2016). Association of Serum Lipids and Coronary Heart Disease in Contemporary Observational Studies. Circulation.

[187] Fox ER, Samdarshi TE, Musani SK (2016). Development and Validation of Risk Prediction Models for Cardiovascular Events in Black Adults: The Jackson Heart Study Cohort. JAMA Cardiol.

[188] Rodriguez F, Chung S, Blum MR, Coulet A, Basu S, Palaniappan LP. (2019). Atherosclerotic Cardiovascular Disease Risk Prediction in Disaggregated Asian and Hispanic Subgroups Using Electronic Health Records. J Am Heart Assoc.

[189] Flores Rosario K, Mehta A, Ayers C (2021). Performance of the Pooled Cohort Equations in Hispanic Individuals Across the United States: Insights From the Multi-Ethnic Study of Atherosclerosis and the Dallas Heart Study. J Am Heart Assoc.

[190] Virtanen M, Nyberg ST, Batty GD (2013). Perceived job insecurity as a risk factor for incident coronary heart disease: systematic review and meta-analysis. BMJ.

[191] Kivimaki M, Nyberg ST, Batty GD (2012). Job strain as a risk factor for coronary heart disease: a collaborative meta-analysis of individual participant data. Lancet.

[192] Kivimaki M, Jokela M, Nyberg ST (2015). Long working hours and risk of coronary heart disease and stroke: a systematic review and meta-analysis of published and unpublished data for 603,838 individuals. Lancet.

[193] Hippisley-Cox J, Coupland C, Robson J, Brindle P. (2014). QRISK2 validation by ethnic group. Heart.

[194] Mehta A, Virani SS, Ayers CR (2020). Lipoprotein(a) and Family History Predict Cardiovascular Disease Risk. J Am Coll Cardiol.

[195] Makshood M, Joshi PH, Kanaya AM (2020). Lipoprotein (a) and aortic valve calcium in South Asians compared to other race/ethnic groups. Atherosclerosis.

[196] Volgman AS, Palaniappan LS, Aggarwal NT (2018). Atherosclerotic Cardiovascular Disease in South Asians in the United States: Epidemiology, Risk Factors, and Treatments: A Scientific Statement From the. American Heart Association. Circulation..

[197] (July 5, 2021). QRISK2-2017.

[198] Brindle P, May M, Gill P (November 2006). Primary prevention of cardiovascular disease: a web-based risk score for seven British black and minority ethnic groups. Heart.

[199] Blaha MJ, DeFilippis AP. (2021). Multi-Ethnic Study of Atherosclerosis (MESA). J Am Coll Cardiol.

[200] Okwuosa TM, Soliman EZ, Lopez F, Williams KA, Alonso A, Ferdinand KC. (2015). Left ventricular hypertrophy and cardiovascular disease risk prediction and reclassification in blacks and whites: The Atherosclerosis Risk in Communities Study. Am Heart J.

[201] Ferdinand KC, Maraboto C. (2019). Is Electrocardiography-Left Ventricular Hypertrophy an Obsolete Marker for Determining Heart Failure Risk With Hypertension?. J Am Heart Assoc.

[202] Nasir K, Shaw LJ, Liu ST (2007). Ethnic differences in the prognostic value of coronary artery calcification for all-cause mortality. J Am Coll Cardiol.

[203] Detrano R, Guerci AD, Carr JJ (2008). Coronary calcium as a predictor of coronary events in four racial or ethnic groups. N Engl J Med.

[204] Frank AT, Zhao B, Jose PO, Azar KM, Fortmann SP, Palaniappan LP. (2014). Racial/ethnic differences in dyslipidemia patterns. Circulation.

[205] Manjunath L, Chung S, Li J, Shah H, Palaniappan L, Yong CM. (2020). Heterogeneity of Treatment and Outcomes Among Asians With Coronary Artery Disease in the United States. J Am Heart Assoc.

[206] Centers for Disease Control and Prevention. Minority Health (April 4, 2021). http://www.cdc.gov/minorityhealth/populations/REMP/aian.html.

[207] (August 6, 2020). Products - Data Briefs - Number 372- August 2020.

[208] Khot UN, Khot MB, Bajzer CT (2003). Prevalence of conventional risk factors in patients with coronary heart disease. JAMA.

[209] Redberg RF, Vogel RA, Criqui MH, Herrington DM, Lima JA, Roman MJ. (2003). 34th Bethesda Conference: Task force #3–What is the spectrum of current and emerging techniques for the noninvasive measurement of atherosclerosis?. J Am Coll Cardiol.

[210] Hiatt WR. (2001). Medical treatment of peripheral arterial disease and claudication. N Engl J Med.

[211] Weatherley BD, Nelson JJ, Heiss G, Chambless LE, Sharrett AR, Nieto FJ (2007). The association of the ankle-brachial index with incident coronary heart disease: the Atherosclerosis Risk In Communities (ARIC) study, 1987–2001. BMC Cardiovasc Disord.

[212] Newman AB, Siscovick DS, Manolio TA, Polak J, Fried LP, Borhani NO (1993). Ankle-arm index as a marker of atherosclerosis in the Cardiovascular Health Study. Circulation.

[213] Moyer VA, U.S. Preventive Services Task Force (2013). Screening for peripheral artery disease and cardiovascular disease risk assessment with the ankle-brachial index in adults: U.S. Preventive Services Task Force recommendation statement. Ann Intern Med.

[214] Fowkes FG, Murray GD, Butcher I (2008). Ankle Brachial Index Collaboration. Ankle brachial index combined with Framingham Risk Score to predict cardiovascular events and mortality: a meta-analysis. JAMA.

[215] Yeboah J, McClelland RM, Polonsky TS (2012). Comparison of Novel Risk Markers for Improvement in Cardiovascular Risk Assessment in Intermediate-Risk Individuals. JAMA.

[216] Goff DC, Lloyd-Jones DM, Bennett G (2014). 2013 ACC/AHA guideline on the assessment of cardiovascular risk: a report of the American College of Cardiology/American Heart Association Task Force on Practice Guidelines. J Am Coll Cardiol.

[217] Gepner AD, Young R, Delaney JA (2015). Comparison of coronary artery calcium presence, carotid plaque presence, and carotid intima-media thickness for cardiovascular disease prediction in the multi-ethnic study of atherosclerosis. Circ Cardiovasc Imaging.

[218] Nambi V, Chambless L, Folsom AR (2010). Carotid intima-media thickness and presence or absence of plaque improves prediction of coronary heart disease risk: the ARIC (Atherosclerosis Risk In Communities) study. J Am Coll Cardiol.

[219] Visseren FLJ, Mach F, Smulders YM (2021). ESC Scientific Document Group; ESC National Cardiac Societies. 2021 ESC Guidelines on cardiovascular disease prevention in clinical practice. Eur Heart J.

[220] Postley JE, Luo Y, Wong ND, Gardin JM. (2015). Identification by ultrasound evaluation of the carotid and femoral arteries of high-risk subjects missed by three validated cardiovascular disease risk algorithms. Am J Cardiol.

[221] Ahmadi N, Nabavi V, Nuguri V (2009). Low fingertip temperature rebound measured by digital thermal monitoring strongly correlates with the presence and extent of coronary artery disease diagnosed by 64-slice multi-detector computed tomography. Int J Cardiovasc Imaging.

[222] Greenland P, Blaha MJ, Budoff MJ, Erbel R, Watson KE. (2018). Coronary Calcium Score and Cardiovascular Risk. J Am Coll Cardiol.

[223] Golub I, Lakshmanan S, Dahal S, Budoff MJ. (2021). Utilizing coronary artery calcium to guide statin use. Atherosclerosis.

[224] Al Rifai M, Blaha MJ, Nambi V (2022). Determinants of Incident Atherosclerotic Cardiovascular Disease Events Among Those With Absent Coronary Artery Calcium: Multi-Ethnic Study of Atherosclerosis. Circulation.

[225] Mahase E. (2021). US taskforce advises against low dose aspirin for primary prevention of cardiovascular disease. BMJ.

[226] Miedema MD, Duprez DA, Misialek JR (2014). Use of Coronary Artery Calcium Testing to Guide Aspirin Utilization for Primary Prevention: Estimates From the Multi-Ethnic Study of Atherosclerosis. Circ Cardiovasc Qual Outcomes.

[227] Michos ED, Blaha MJ, Blumenthal RS. (2017). Use of the Coronary Artery Calcium Score in Discussion of Initiation of Statin Therapy in Primary Prevention. Mayo Clin Proc.

[228] Malik S, Budoff MJ, Katz R (2011). Impact of subclinical atherosclerosis on cardiovascular disease events in individuals with metabolic syndrome and diabetes: the multi-ethnic study of atherosclerosis. Diabetes Care.

[229] Mortensen MB, Blaha MJ. (2021). Is There a Role of Coronary CTA in Primary Prevention? Current State and Future Directions. Curr Atheroscler Rep.

[230] Budoff MJ, Lakshmanan S, Toth PP (2022). Cardiac CT angiography in current practice: An American society for preventive cardiology clinical practice statement. Am J Prev Cardiol.

[231] Mulcahy R, Hickey N, Graham I, McKenzie G (1974). Factors influencing long-term prognosis in male patients surviving a first coronary attack. Br Heart J.

[232] Colantonio LD, Shannon ED, Orroth KK (2019). Ischemic Event Rates in Very-High-Risk Adults. J Am Coll Cardiol.

[233] Vallejo-Vaz AJ, Ray KK, Ginsberg HN (2019). Associations between lower levels of low-density lipoprotein cholesterol and cardiovascular events in very high-risk patients: Pooled analysis of nine ODYSSEY trials of alirocumab versus control. Atherosclerosis.

[234] Piepoli MF, Hoes AW, Agewall S (2016). 2016 European Guidelines on cardiovascular disease prevention in clinical practice: The Sixth Joint Task Force of the European Society of Cardiology and Other Societies on Cardiovascular Disease Prevention in Clinical Practice. Eur Heart J.

[235] Mach F, Baigent C, Catapano AL, Koskinas KC, Casula M, Badimon L (2020). 2019 ESC/EAS Guidelines for the management of dyslipidaemias: lipid modification to reduce cardiovascular risk. The Task Force for the management of dyslipidaemias of the European Society of Cardiology (ESC) and European Atherosclerosis Society (EAS). Eur Heart J.

[236] Virani SS, Smith SC, Stone NJ, Grundy SM (2020). Secondary Prevention for Atherosclerotic Cardiovascular Disease. Comparing Recent US and European Guidelines on Dyslipidemia. Circulation.

[237] Kotseva K, De Backer G, De Bacquer D (2019). on behalf of the EUROASPIRE Investigators. Lifestyle and impact on cardiovascular risk factor control in coronary patients across 27 countries: results from the European Society of Cardiology ESC-EORP EUROASPIRE V registry. Eur J Prev Cardiol.

[238] Zhao M, Marie Cooney MT, Klipstein-Grobusch K (2016). Simplifying the audit of risk factor recording and control: A report from an international study in 11 countries. Eur J Prev Cardiol.

[239] Karlson BW, Wiklund O, Palmer MK, Nicholls SJ, Lundman P, Barter PJ. (2016). Variability of low-density lipoprotein cholesterol response with different doses of atorvastatin, rosuvastatin, and simvastatin: results from VOYAGER. Eur Heart J Cardiovasc Pharmacother.

[240] De Bacquer D, Ueda P, Reiner Z (2020). for the EUROASPIRE IV and V National Coordinators. Prediction of recurrent event in patients with coronary heart disease: the EUROASPIRE Risk Model- Results from a prospective study in 27 countries in the WHO European region - The EURObservational Research Programme (EORP) of the European Society of Cardiology (ESC). Eur J Prev Cardiol.

[241] Dorresteijn JAN, Visseren FLJ, Wassink AMJ (2013). on behalf of the SMART Study Group. Development and validation of a prediction rule for recurrent vascular events based on a cohort study of patients with arterial disease: the SMART risk score. Heart.

[242] Wilson PWF, D'Agostino R, Bhatt DL, Registry REACH (2012). An international model to predict recurrent cardio- vascular disease. Am J Med.

